# Adverse outcomes among pregnant women with COVID‐19 according to hospitalization status: A prospective individual participant data meta‐analysis in Europe and North America

**DOI:** 10.1002/ijgo.70694

**Published:** 2025-12-15

**Authors:** Odette de Bruin, Emeline Maisonneuve, Eimir Hurley, Hedvig M. E. Nordeng, Anick Bérard, Odile Sheehy, Padma Kaul, Mayura U. Shinde, Austin Cosgrove, Jennifer G. Lyons, Elizabeth Messenger‐Jones, Maria E. Kempner, Sengwee Toh, Wei Hua, José J. Hernández‐Muñoz, Leyla Sahin, Carolyn E. Cesta, David Hägg, Rosa Gini, Olga Paoletti, Beatriz Poblador‐Plou, Sue Jordan, Clara L. Rodríguez‐Bernal, Francisco Sánchez‐Sáez, Régis Lassalle, Marie‐Agnès Bernard, Fariba Ahmadizar, Guillaume Favre, Alice Panchaud, Kitty W. M. Bloemenkamp, Kelly Plueschke, Corinne de Vries, Satu J. Siiskonen, Miriam C. J. M. Sturkenboom, the CONSIGN Collaboration Group, Benjamin P. Geisler, Mark Walker, Steven Hawken, Sasha Bernatsky, Sherif Eltonsy, Emma Hoffman, Andrew B. Petrone, Jolene Mosley, Jenice Ko, Claudia Bartolini, Giuseppe Roberto, Giorgio Limoncella, Anna Girardi, Giulia Hyeraci, Antonio Gimeno‐Miguel, Jonás Carmona‐Pírez, Antonio Poncel‐Falcó, Aida Moreno‐Juste, Alexandra Prados‐Torres, Daniel Thayer, Ian Farr, Saira Ahmed, Ieuan Scanlon, Gabriel Sanfélix‐Gimeno, Isabel Hurtado, Anibal Garcia‐Sempere, Salvador Peiro, Jérémy Jové, Dunia Sakr, Cécile Droz‐Perroteau, Ema Alsina, David Baud, Hilde M. Engjom, Judit Riera‐Arnau, Mònica Sabaté Gallego, Elena Ballarín Alins, Cristina Aguilera Martin, Melissa Kampman, Celline Brasil

**Affiliations:** ^1^ Department of Data Science & Biostatistics, Julius Global Health University Medical Center Utrecht (UMCU) Utrecht The Netherlands; ^2^ Division Woman and Baby, Department of Obstetrics, Wilhelmina Children's Hospital University Medical Center Utrecht (UMCU) Utrecht The Netherlands; ^3^ Institute of Primary Health Care (BIHAM) University of Bern Bern Switzerland; ^4^ Graduate School for Health Sciences (GHS) University of Bern Bern Switzerland; ^5^ Materno‐Fetal and Obstetrics Research Unit, Woman‐Mother‐Child Department Lausanne University Hospital Lausanne Switzerland; ^6^ Pharmacoepidemiology and Drug Safety Research Group, Department of Pharmacy University Oslo (UiO) Oslo Norway; ^7^ Department of Child Health and Development Norwegian Institute of Public Health Oslo Norway; ^8^ Faculty of Pharmacy University of Montreal Montreal Quebec Canada; ^9^ Research Center Center Hospitalier Universitaire (CHU) de Sainte‐Justine Montreal Quebec Canada; ^10^ Department of Medicine University of Alberta Edmonton Alberta Canada; ^11^ Department of Population Medicine Harvard Medical School and Harvard Pilgrim Health Care Institute Boston Massachusetts USA; ^12^ Department of Population Medicine Harvard Pilgrim Health Care Institute Boston Massachusetts USA; ^13^ Office of Surveillance and Epidemiology, Center for Drug Evaluation and Research U.S. Food and Drug Administration Silver Spring Maryland USA; ^14^ Office of New Drugs, Center for Drug Evaluation and Research U.S. Food and Drug Administration Silver Spring Maryland USA; ^15^ Department of Medicine Solna, Center for Pharmacoepidemiology Karolinska Institutet Stockholm Sweden; ^16^ Epidemiology Unit Tuscan Regional Healthcare Agency Florence Italy; ^17^ EpiChron Research Group, Aragon Health Sciences Institute (IACS), IIS Aragón Miguel Servet University Hospital Zaragoza Spain; ^18^ Network for Research on Chronicity, Primary Care and Health Promotion (RICAPPS), Research Network on Health Services in Chronic Diseases Institute of Health Carlos III Madrid Spain; ^19^ Faculty of Medicine, Health and Life Science Swansea University Swansea UK; ^20^ Health Services Research and Pharmacoepidemiology Unit Foundation for the Promotion of Health and Biomedical Research of Valencia Region Valencia Spain; ^21^ Bordeaux PharmacoEpi, INSERM CIC‐P1401 Université de Bordeaux Bordeaux France; ^22^ Service of Pharmacy Lausanne University Hospital and University of Lausanne Lausanne Switzerland; ^23^ European Medicines Agency Amsterdam The Netherlands; ^24^ Division of Pharmacoepidemiology and Clinical Pharmacology, Utrecht Institute for Pharmaceutical Sciences (UIPS) Utrecht University Utrecht The Netherlands

**Keywords:** adverse outcomes, COVID‐19, hospitalization, international collaboration, meta‐analysis, pregnancy

## Abstract

**Background:**

Understanding the varied impact of COVID‐19 severity on pregnancy outcomes is crucial for informed clinical management and targeted interventions.

**Objective:**

To evaluate the impact of COVID‐19 on pregnancy outcomes, distinguishing between pregnant women managed in primary care and those requiring hospitalization.

**Search Strategy:**

Regulatory authorities actively promoted global cooperation on COVID‐19's impact during pregnancy. Data were obtained through these regulatory bodies and direct researcher communication rather than through systematic searches.

**Selection Criteria:**

Data sources required secondary population‐based data to identify pregnancies with COVID‐19, along with hospitalization, diagnostic and medication codes. Eligibility for the meta‐analysis was determined through protocol evaluation and researcher consultations.

**Data Collection and Analysis:**

PRISMA‐IPD and Cochrane guidelines for prospective meta‐analysis were followed. Protocols and definitions were standardized across sources, and a common R script was developed. Initially, crude and adjusted relative risks (aRR) with 95% confidence intervals (CI) were calculated to assess adverse outcomes in pregnant women with and without COVID‐19 in each data source. Estimates were stratified by trimester at infection and hospitalization status. Subsequently, data were pooled using a random‐effects meta‐analysis.

**Main Results:**

Data from 10 sources across seven countries contributed to the meta‐analysis, including 86 210 pregnant women diagnosed with COVID‐19, of whom 4.4% were hospitalized. Non‐hospitalized pregnant women with COVID‐19 had no increased risks of adverse outcomes compared to pregnant women without COVID‐19. However, hospitalized women with COVID‐19 in each trimester had higher risks of cesarean section, preterm birth, and LBW compared to pregnant women without COVID‐19. Hospitalization due to COVID‐19 in the third trimester was associated with increased risk of stillbirth (aRR 5.90, 95% CI: 2.22–15.71, *I* ^2^ = 0%). First‐trimester hospitalizations due to COVID‐19 did not show heightened risks of GDM (aRR 2.08, 95% CI: 0.93–4.64, *I* ^2^ = 65%), pre‐eclampsia (aRR 1.79, 95% CI: 0.48–6.66, *I* ^2^ = 71%), or major congenital anomalies (aRR 1.30, 95% CI: 0.55–3.06, *I* ^2^ = 0%).

**Conclusions and Relevance:**

COVID‐19 requiring hospitalization is associated with adverse pregnancy outcomes, emphasizing the need to prevent severe illness during pregnancy. This study also highlights the importance of international collaboration for gathering pregnancy data and shows that building global research networks is essential for responding to future health crises.

## 1 INTRODUCTION

The COVID‐19 pandemic brought unprecedented challenges to global healthcare systems, profoundly affecting most facets of public health.^1^ Among the populations significantly affected by this virus were pregnant women.^2^ As the pandemic unfolded, emerging evidence pointed towards heightened risks and complications associated with COVID‐19 during pregnancy.^3^, ^4^ Understanding the implications of COVID‐19 on pregnancy‐related outcomes is essential for informed clinical decision making and development of targeted interventions. However, the variability in disease severity among affected women poses a significant challenge to comprehensively assessing the impact on maternal and fetal outcomes.

While existing studies have shed light on the association between COVID‐19 and adverse pregnancy outcomes, many have not systematically stratified their analyses based on disease severity. COVID‐19 severity was provided for 8.8% of pregnant women included in a systematic review and meta‐analysis from the year 2021.^4^ This omission potentially undermines the accuracy of reported findings and limits the generalizability of conclusions drawn. As COVID‐19 manifests with a wide spectrum of clinical presentations, ranging from asymptomatic or mild to severe respiratory distress requiring hospitalization, the differences in impact on maternal and fetal health outcomes becomes increasingly apparent.^5^

Recognizing this gap in knowledge, our international collaborative effort, the COVID‐19 infection and medicines in pregnancy (CONSIGN) project, undertaken within the EU Pharmacoepidemiology and Pharmacovigilance (PE&PV) Research Network and funded by the European Medicines Agency (EMA), aimed at elucidating the relationship between COVID‐19 severity and pregnancy‐related adverse outcomes. Within the CONSIGN project, we conducted a prospective two‐stage individual participant data (IPD) meta‐analysis of studies with similar protocols and settings that utilized secondary data.^6^, ^7^, ^8^ Our objective was to assess the impact of COVID‐19 on pregnancy‐related outcomes, distinguishing between pregnant women managed in primary care and those needing hospital admission.

## 2 METHODS

We conducted a prospective two‐stage IPD meta‐analysis, combining findings from various data sources utilizing secondary administrative and electronic health record (EHR) data or medical claims data. The study is registered in the HMA‐EMA catalog of real‐world data studies with the identifier EUPAS40317, and the protocol and statistical analysis plan (SAP) are available online.^8^ We adhered closely to PRISMA‐IPD and Cochrane guidelines for prospective meta‐analysis.^9^, ^10^

### 2.1 Study selection processes

EMA and CONSIGN leadership, in collaboration with the International Coalition of Medicines Regulatory Authorities (ICMRA), engaged with the U.S. Food and Drug Administration (FDA) and Health Canada to promote international cooperation on research on COVID‐19 in pregnancy.^11^, ^12^ Data sources were accessed through regulatory authorities and direct communication among researchers, rather than with a systematic search.^6^, ^7^ Eligibility criteria included access to population‐based data capable of identifying start and end dates of pregnancies, and linking them to COVID‐19 records, hospitalization and diagnostic codes, and medication use. Compliance with the CONSIGN EHR study protocol (EUPAS39438) was mandatory, either fully or partially.^6^, ^7^, ^8^, ^13^, ^14^ Data sources had to provide SAPs and additional materials for review, followed by meetings to assess whether they met the eligibility criteria for the meta‐analysis.

### 2.2 Data items

To standardize data semantics for this meta‐analysis, we reviewed protocols, codebooks, and definitions of populations, exposures, outcomes, and covariates across data sources. The study population comprised pregnant women diagnosed with COVID‐19 during pregnancy from January 2020 to the latest available data from each data source. Whenever feasible within the data source, this exposed cohort was appropriately matched to a comparator cohort of pregnant women without COVID‐19 based on pregnancy trimester at infection (pregnancy start within ±14 days of exposed cohort), calendar month of COVID‐19 infection, and maternal age. If matched, uninfected women were assigned the index date of the corresponding COVID‐19 diagnosis in the matched individual.

Outcomes of interest were categorized into maternal, pregnancy, and neonatal outcomes that included: maternal death, gestational diabetes (GDM), pre‐eclampsia, cesarean section, preterm birth, stillbirth, neonatal death, low birth weight (LBW), small for gestational age (SGA), and major congenital anomalies (Table S2).^15^, ^16^, ^17^, ^18^, ^19^, ^20^, ^21^, ^22^, ^23^ Covariates of interest encompassed maternal age, trimester of pregnancy, calendar month of COVID‐19 diagnosis, hospitalization status, medical conditions associated with severe COVID‐19, and conditions associated with obstetric complications (Table S3). Non‐hospitalized women had a positive COVID‐19 test or diagnosis without requiring hospitalization for COVID‐19 within four weeks. Hospitalized women had a COVID‐19 positive test or complication recorded in hospital diagnostic fields. However, those diagnosed with COVID‐19 near delivery or hospitalization for obstetric reasons, without codes indicating severe symptoms, were excluded.

### 2.3 Data collection process

A common R‐script, aligned with the ConcePTION common data model (CDM) structure, was developed for data sources participating in the CONSIGN EHR study, with the exception of Karolinska Institutet in Sweden, which developed SAS script based on the common R‐script for their analysis.^24^ This common R‐script, along with the SAP, code lists for outcomes and exposures, covariates, and definitions of risk windows, was distributed to all other eligible data sources. They utilized this material to locally generate results, either through R or SAS. Each data source provided aggregated results, including counts, proportions, and effect estimates, in predefined shell tables.

### 2.4 Synthesis methods

Initially, we computed crude and adjusted relative risk (RR) with 95% confidence intervals (CI) for adverse outcomes in pregnant women with COVID‐19 and without COVID‐19 at each study site. Adjustments were made for age, medical conditions associated with severe COVID‐19, and conditions associated with obstetric complications. Table S4 summarizes the main variations between study sites. Analyses were stratified by pregnancy trimester at the time of COVID‐19 infection and hospitalization status.

Subsequently, meta‐analyses were conducted using R software, version 4.2.2, with the metafor package. A random‐effects meta‐analysis of aRRs was performed using the rma function, enabling the calculation of a combined effect estimate with a corresponding 95% CI. The restricted maximum likelihood (REML) method was employed for model estimation.^25^, ^26^

If a data source had small sample sizes and/or low event rates, reliable estimates could not be computed, leading to exclusion from the meta‐analysis; however, these numbers are reported in the Supporting Information Tables. Forest plots depicted individual site estimates (with 95% CI) for each data source, with a diamond representing the pooled point estimate (95% CI) for each outcome of interest. Heterogeneity was assessed through visual examination of the forest plot and the *I* ^2^ statistic (40% or greater).

## 3 RESULTS

### 3.1 Study site selection

**FIGURE 1:**
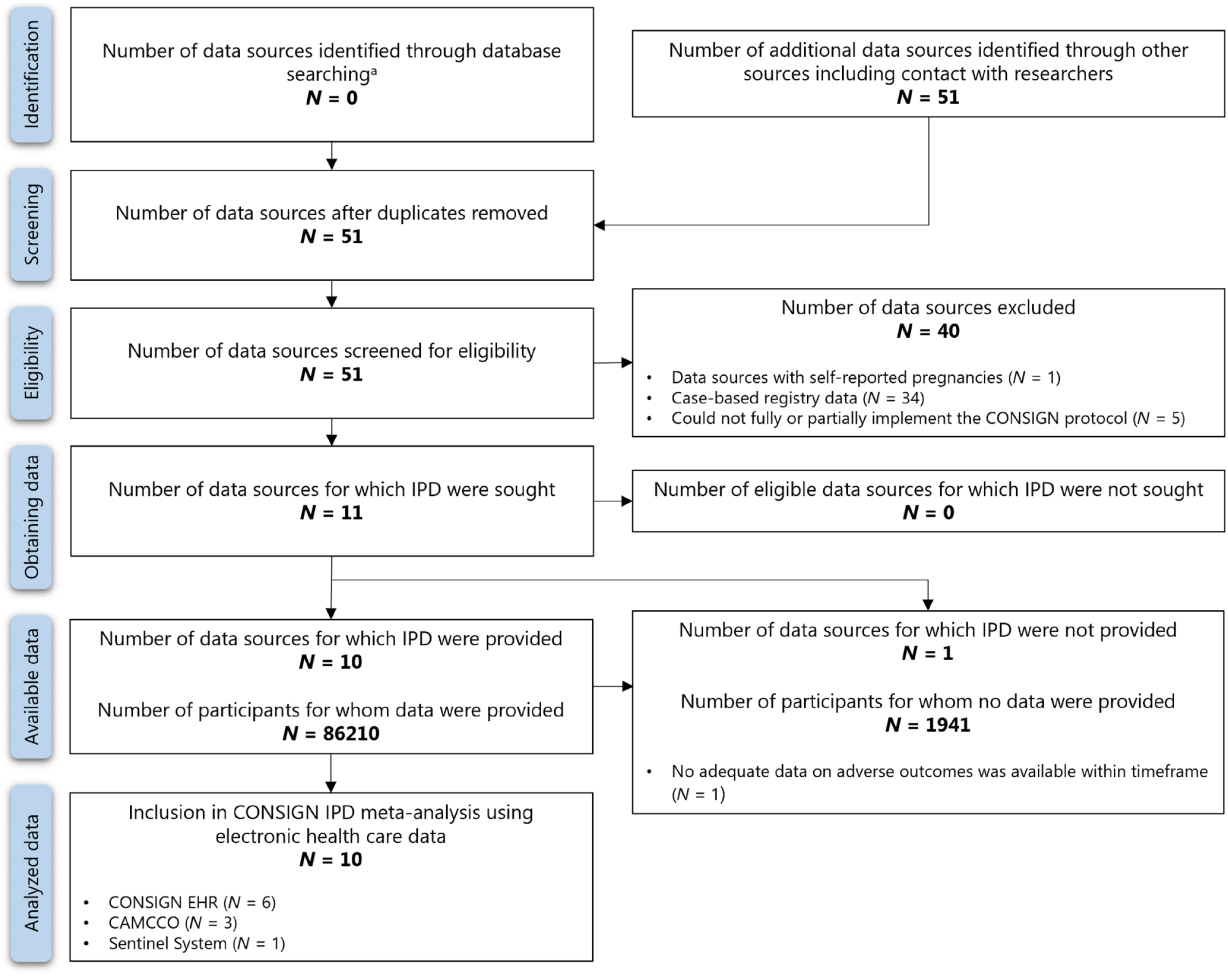
Flow chart of the selection process of networks and data sources participating in the individual participant data (IPD) meta‐analysis using secondary healthcare data. Adapted from the PRISMA IPD flow diagram. CAMCCO, Canadian Mother–Child Cohort; EHR, electronic health record; IPD, individual participant data. ^a^No systematic search was performed; instead, data sources were accessed through regulatory authorities and direct communication among researchers.

We identified 51 networks and data sources with data on pregnant women with COVID‐19, of which 16 utilized secondary data. Five of these 16 data sources were unable to implement the CONSIGN EHR protocol and were excluded.^6^, ^7^ Another data source was excluded during the analysis stage due to inadequate data on the adverse outcomes of interest (Figure 1). Ultimately, results from 10 data sources across seven countries, accessible through three research initiatives, were included in the meta‐analysis, with their characteristics detailed in Table S5.

### 3.2 Study characteristics

The CONSIGN EHR study incorporated data from six electronic healthcare registries spanning five European countries (Tuscany, Italy; France; Valencia, Spain; Aragon, Spain; Norway; Sweden).^13^, ^14^ The Canadian Mother–Child Cohort (CAMCCO) Active Surveillance Initiative gathered data from three provinces, Alberta, Manitoba, and Ontario, with results presented separately.^27^, ^28^ The U.S. FDA Sentinel System utilized the most recent data from seven data partners participating in the Rapid COVID‐19 Sentinel Distributed Database, including four national health insurers and three regional integrated care delivery systems (Table S5).^12^, ^29^, ^30^

Across all sites, the start of a pregnancy was defined as the estimated first day of the last menstrual period (LMP), while the end of a pregnancy was defined as the date of birth (or non‐live pregnancy end in sites collecting this information) (Table S6). The Sentinel System pregnancy algorithm for this analysis identified only live births. Trimester of pregnancy definitions varied slightly among the three included networks (Table S3). In all data sources, confirmed COVID‐19 diagnoses were identifiable through PCR or antigen tests, except in France where they were based on diagnostic codes only (Table S6).

### 3.3 Description of the cohort

In total, 86 210 pregnant women diagnosed with COVID‐19 during their pregnancy were identified (Table 1). Nearly half of these cases (49%) occurred during the third trimester. The distribution of COVID‐19 infections across the pregnancy trimesters varied considerably across regions. France, Sweden, and Manitoba‐Canada had fewer first trimester diagnosed infections than other regions (Table 1). Of the 86 210 pregnant women, 3792 (4.4%) were hospitalized for COVID‐19. Notably, France had access to hospitalized patient data only, while Ontario‐Canada had no recorded hospitalized cases (Table 1). Figure S1 displays the timing of COVID‐19 infection in the pregnant cohort from December 2019 to December 2022, categorized by data source. Most countries provided data for 2020 and 2021; the US data extended until the end of 2022; however, France and Sweden had data only for 2020 (Table S5). Baseline characteristics of pregnant women with and without COVID‐19 are detailed in Table S7.

**TABLE 1:** Description of the pregnancy cohort who tested positive for COVID‐19 by pregnancy trimester of infection and hospitalization status.

	Total number of pregnant women with COVID‐19	COVID‐19 in trimester 1	COVID‐19 in trimester 2	COVID‐19 in trimester 3
	No.	No. (%)	No. (%)	No. (%)
Tuscany, Italy	995	207 (21)	278 (28)	510 (51)
France	1069	7 (0.7)	92 (9)	970 (91)
Valencia, Spain	3654	1278 (35)	1047 (29)	1329 (36)
Aragon, Spain	951	209 (22)	284 (30)	458 (48)
Norway	1146	235 (21)	409 (36)	502 (44)
Sweden	5030	290 (6)	1711 (34)	3029 (60)
Alberta, Canada	2296	580 (25)	741 (32)	975 (43)
Manitoba, Canada	235	19 (8)	62 (26)	154 (66)
Ontario, Canada	933	270 (29)	303 (32)	360 (39)
USA	69 901	15 841 (23)	20 121 (29)	33 979 (49)
Total	86 210	18 936 (22)	25 048 (29)	42 266 (49)

	Number of non‐hospitalized pregnant women with COVID‐19^b^	COVID‐19 in trimester 1	COVID‐19 in trimester 2	COVID‐19 in trimester 3
	No.	No. (%)	No. (%)	No. (%)
Tuscany, Italy	739	181 (24)	261 (35)	297 (40)
France^a^	0	0 (0)	0 (0)	0 (0)
Valencia, Spain	3231	1241 (38)	1032 (32)	958 (30)
Aragon, Spain	754	201 (27)	272 (36)	281 (37)
Norway	879	217 (25)	361 (41)	301 (34)
Sweden	4199	259 (6)	1455 (35)	2485 (59)
Alberta, Canada	1968	571 (29)	706 (36)	691 (35)
Manitoba, Canada	170	19 (11)	62 (36)	89 (52)
Ontario, Canada	933	270 (29)	303 (32)	360 (39)
USA	68 041	15 774 (23)	19 884 (29)	32 503 (48)
Total	80 914	18 733 (23)	24 336 (30)	37 965 (47)

	Number of hospitalized pregnant women with COVID‐19^b^	COVID‐19 in trimester 1	COVID‐19 in trimester 2	COVID‐19 in trimester 3
	No.	No. (%)	No. (%)	No. (%)
Tuscany, Italy	133	9 (7)	17 (13)	107 (80)
France	323	7 (2)	92 (28)	224 (69)
Valencia, Spain	33	13 (39)	12 (36)	8 (24)
Aragon, Spain	26	3 (12)	12 (46)	11 (42)
Norway	193	18 (9)	48 (25)	127 (66)
Sweden	831	31 (4)	256 (31)	544 (65)
Alberta, Canada	328	9 (3)	35 (11)	284 (87)
Manitoba, Canada	65	0 (0)	0 (0)	65 (100)
Ontario, Canada	0	0 (0)	0 (0)	0 (0)
USA	1860	67 (4)	237 (13)	1472 (79)
Total	3792	157 (4)	709 (19)	2842 (75)

^a^ COVID‐19 cases in France were identified through hospital admissions records with a COVID‐19 diagnosis. Consequently, pregnant women not requiring hospitalization are not included.

^b^ Cases in which the COVID‐19 test/positive diagnosis was ±2 days of delivery date did not contribute to analyses by hospitalization. They did however contribute to the “total cases.” Therefore, in some sites hospitalized and non‐hospitalized cases do not add up to 100%.

### 3.4 Maternal outcomes

**FIGURE 2:**
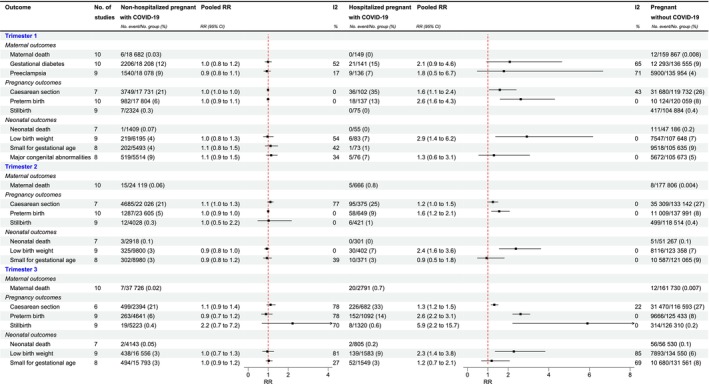
Prevalence of adverse maternal, pregnancy and neonatal outcomes and associations with COVID‐19 by trimester at infection and hospitalization status.

The prevalence of maternal deaths in each trimester was low, precluding a reliable meta‐analysis (Figure 2). There was no evidence indicating COVID‐19 infection in the first trimester is associated with an increased risk of gestational diabetes for either non‐hospitalized (aRR 0.99, 95% CI: 0.84–1.17, *I* ^2^ = 52%) or hospitalized pregnant women with COVID‐19 (aRR 2.08, 95% CI: 0.93–4.64, *I* ^2^ = 65%) (Figure 2). Similarly, there was no evidence suggesting COVID‐19 infection in the first trimester is associated with an increased risk of pre‐eclampsia for either non‐hospitalized (aRR 0.93, 95% CI: 0.78–1.12, *I* ^2^ = 17%) or hospitalized pregnant women with COVID‐19 (aRR 1.79, 95% CI: 0.48–6.66, *I* ^2^ = 71%) (Figure 2). The corresponding forest plots are shown in Figures S2 and S3, and the prevalence rates from the different data sources are displayed in Tables S8–S10.

### 3.5 Pregnancy outcomes

There was no increased risk of cesarean section among non‐hospitalized pregnant women with COVID‐19 in any trimester, compared to those without COVID‐19, with aRR 0.99 (95% CI: 0.97–1.02, *I* ^2^ = 0%) for the first, aRR 1.11 (95% CI: 0.97–1.26, *I* ^2^ = 77%) for the second and aRR 1.12 (95% CI: 0.93–1.36, *I* ^2^ = 78%) for the third trimester. However, hospitalized pregnant women with COVID‐19 in any trimester had an increased risk of cesarean section compared with pregnant women without COVID‐19, with aRR 1.59 (95% CI: 1.06–2.40, *I* ^2^ = 43%) for the first, aRR 1.25 (95% CI: 1.04–1.50, *I* ^2^ = 0%) for the second, and aRR 1.32 (95% CI: 1.16–1.51, *I* ^2^ = 22%) for the third trimester (Figure 2). There was no evidence that COVID‐19 infection in any trimester increased the risk of preterm birth in non‐hospitalized pregnant women with COVID‐19, compared to those without COVID‐19, with aRR 0.99 (95% CI: 0.94–1.06, *I* ^2^ = 0%), aRR 1.00 (95% CI: 0.95–1.05, *I* ^2^ = 0%), and aRR 0.89 (95% CI: 0.66–1.21, *I* ^2^ = 78%), respectively. However, hospitalized pregnant women with COVID‐19 in any trimester had a higher risk of preterm birth compared with pregnant women without COVID‐19, with aRR 2.62 (95% CI: 1.60–4.29, *I* ^2^ = 0%), aRR 1.56 (1.18–2.07, *I* ^2^ = 0%), and aRR 2.61 (95% CI: 2.22–3.07, *I* ^2^ = 0%), respectively (Figure 2).

The overall rate of stillbirth was low. Among non‐hospitalized pregnant women with COVID‐19 in the second trimester, compared with pregnant women without COVID‐19, there was no increased risk of stillbirth (aRR 1.02, 95% CI: 0.48–2.17, *I* ^2^ = 0%). In the third trimester, there was no evidence suggesting an increased risk of stillbirth among non‐hospitalized pregnant women with COVID‐19 compared to pregnant women without COVID‐19 (aRR 2.22, 95% CI: 0.69–7.17, *I* ^2^ = 70%). However, compared to pregnant women without COVID‐19, hospitalized pregnant women with COVID‐19 in the third trimester had an increased risk of stillbirth (aRR 5.90, 95% CI: 2.22–15.71, *I* ^2^ = 0%) (Figure 2). The corresponding forest plots are shown in Figures S4–S11, and the prevalence rates from the different data sources are displayed in Tables S11–S13.

### 3.6 Neonatal outcomes

The prevalence of neonatal deaths in each trimester was low, precluding a reliable meta‐analysis (Figure 2). COVID‐19 infection during pregnancy did not increase the risk of LBW in neonates from non‐hospitalized pregnant women with COVID‐19 compared to those without COVID‐19 for each trimester, aRR 0.97 (95% CI: 0.76–1.26, *I* ^2^ = 54%), aRR 0.93 (95% CI: 0.82–1.05, *I* ^2^ = 0%), and aRR 0.96 (95% CI: 0.72–1.28, *I* ^2^ = 81%), respectively. However, neonates of hospitalized pregnant women with COVID‐19 had a higher risk of LBW in each trimester, aRR 2.92 (95% CI: 1.38–6.17, *I* ^2^ = 0%), aRR 2.38 (1.56–3.61, *I* ^2^ = 0%), and aRR 2.28 (95% CI: 1.36–3.82, *I* ^2^ = 85%), respectively (Figure 2). No association was found between COVID‐19 infection during pregnancy and SGA in neonates from non‐hospitalized pregnant women, for each trimester, aRR 1.12 (95% CI: 0.85–1.48, *I* ^2^ = 42%), aRR 0.94 (95% CI: 0.76–1.17, *I* ^2^ = 39%) and aRR 1.03 (95% CI: 0.89–1.21, *I* ^2^ = 27%), respectively. In hospitalized pregnant women with COVID‐19, an infection in the second and third trimesters was not associated with an increased risk of SGA in their offspring (aRR 0.94, 95% CI 0.49–1.81, *I* ^2^ = 0%, and aRR 1.19, 95% CI: 0.69–2.07, *I* ^2^ = 69%, respectively) (Figure 2). There was no evidence suggesting that COVID‐19 infection in the first trimester was associated with an increased risk of major congenital anomalies in neonates from both non‐hospitalized (aRR 1.15, 95% CI: 0.91–1.46, *I* ^2^ = 34%) and hospitalized pregnant women (aRR 1.30, 95% CI: 0.55–3.06, *I* ^2^ = 0%) (Figure 2). The corresponding forest plots are shown in Figures S12–S18, and the prevalence rates from the different data sources are displayed in Tables S14–S17.

## 4 DISCUSSION

This international prospective two‐stage IPD meta‐analysis combined data from 10 sources across seven countries, examining adverse outcomes in a substantial cohort of pregnant women with COVID‐19 using the same protocol. The study compared these outcomes to those in pregnant women without COVID‐19, considering trimester at infection and hospitalization status. No increased risk of adverse maternal, pregnancy or neonatal outcomes was observed among non‐hospitalized pregnant women with COVID‐19. However, hospitalized pregnant women with COVID‐19 were more likely to give birth by cesarean section, and had increased risks of preterm birth and LBW, across all trimesters. Additionally, third trimester COVID‐19 infection requiring hospitalization was associated with an increased risk of stillbirth. First trimester COVID‐19 infection requiring hospitalization was not associated with increased risks of gestational diabetes, pre‐eclampsia, and congenital anomalies, although event rates for congenital anomalies were low.

When interpreting this meta‐analysis, several limitations must be considered. First, heterogeneity among the data sources, especially in the non‐hospitalized group, may stem from differences in data collection and harmonization challenges. Despite efforts to align variables and analyses between the CONSIGN EHR study, CAMCCO, and the Sentinel System, complete alignment was difficult to achieve. For instance, we could not adjust for covariates such as socioeconomic status, substance misuse, and smoking, which were inconsistently recorded. Additionally, CAMCCO did not apply matching, but the baseline characteristics were comparable, suggesting minimal impact on the results. The Sentinel System data primarily included commercially insured populations, potentially underrepresenting publicly insured and uninsured persons.^30^ Moreover, Sentinel could only identify pregnancies resulting in live births, possibly favoring low‐risk pregnancies.^29^ Excluding the Sentinel data, however, would significantly reduce the overall sample size as it is the largest dataset.

Another explanation for heterogeneity could be the varying prevalence and impact of COVID‐19 variants across Europe, the US, and Canada. In 2020 and 2021, the dominant variants were Alpha (B.1.1.7) and Delta (B.1.617.2), both highly transmissible, with Delta also showing partial immune escape.^31^ Our data for the US extends into 2022, marked by the emergence of the Omicron variant (B.1.1.529), which generally caused milder illness than Delta.^32^, ^33^, ^34^ These evolving variants, with different transmissibility, severity, and immune escape characteristics, may contribute to the observed differences in COVID‐19 outcomes. Moreover, the lack of information on COVID‐19 vaccination status may restrict the applicability of our findings beyond 2022, considering widespread immunity acquired through infection or vaccination.

Second, although stratifying results by trimester at onset of infection allowed us to inspect impact of infection by trimester, this led to small sample sizes in some data sources, resulting in less precise estimates shown by wide confidence intervals. While not part of the SAP, pooling data from all trimesters could offer more robust findings regarding COVID‐19's overall impact. Furthermore, some data sources could not contribute to the analyses of all adverse outcomes, due to limited access or low event numbers, posing a risk of re‐identification of women and need to mask. This risk also prevented the breakdown of observed malformations, potentially obscuring specific malformation risks within the overall numbers.

A review of 42 studies involving 440 000 pregnant women found increased risks of pre‐eclampsia, preterm birth and stillbirth. Comparable to our results, the risks were significantly greater among those severely affected.^35^ The systematic review and meta‐analysis by Allotey et al. also reported increased risks for cesarean section, preterm birth, stillbirth, and neonatal death, although the overall incidence of stillbirth and neonatal death was relatively low, as in our study.^3^ However, this review did not differentiate between non‐hospitalized and hospitalized cases or between trimesters, and our study found higher risks in the hospitalized group.

The findings highlight the association between severe COVID‐19 and adverse pregnancy outcomes, highlighting the need to prevent severe illness during pregnancy. The reluctance to medicate or vaccinate pregnant women against COVID‐19 may have contributed to these outcomes.^36^, ^37^ Other CONSIGN project studies indicated that medication was rarely used to treat COVID‐19 during pregnancy, and practice has changed over time.^14^, ^38^, ^39^, ^40^ These studies also found that medication administration is closely linked to disease severity, precluding delineation of causal inferences regarding the effect of these medications on adverse outcomes.^38^, ^39^

Our study design enhances the generalizability of results by stratifying the analyses by hospitalization status, thereby increasing applicability to diverse settings. Notably, CONSIGN data sources excluded women testing positive for COVID‐19 within two days of delivery from the hospitalized group to prevent misclassification of disease severity due to routine screening for COVID‐19 at delivery wards. The Sentinel System excluded pregnancies with maternal outcomes before COVID‐19. Due to most third‐trimester COVID‐19 cases occurring around delivery, strict temporality requirements led to high attrition, so estimates for maternal outcomes in this group were not reported.

This international prospective IPD‐meta‐analysis highlights the increased risks of adverse outcomes among pregnant women hospitalized due to COVID‐19, emphasizing the need for preventive measures and comprehensive healthcare strategies. Moving forward, fostering international collaboration in pregnancy research is crucial for gathering data from a larger and diverse population sample. Therefore, in preparation for future pandemics or global health challenges, establishing essential infrastructure, funding mechanisms, and structural frameworks for global collaborative research networks is imperative. These efforts will facilitate rapid and thorough gathering of vital information and data essential for effective response and mitigation strategies.

## AUTHOR CONTRIBUTIONS

The CONSIGN core research team (OdB, EM, EH, HMEN, FA, GF, AP, KWMB, KP, CdV, SJS, and MCJM) drafted the protocol and SAP. The protocol and SAP were revised and finalized based on feedback from all co‐authors. OdB and EM coordinated contact with all data access providers. The following authors had the main responsibility for local data analysis: Tuscany, Italy (RG, OP), France (RL, MAB), Valencia, Spain (CLRB, FSS), Aragon, Spain (BPP), Norway (HMEN), Sweden (CEC, DH), Canada (AB, OS, PK), US (MUS, AC, JGL, EMJ, MEK, ST, WH, JJHM, LS). OdB developed the R‐scripts for meta‐analysis, created the figures and tables for all the results. All authors participated in interpreting the data, reviewing the manuscript, and approved the final version. OdB attests that all listed authors meet authorship criteria and that no others meeting the criteria have been omitted. Each author accepts accountability for their part of the paper as published. OdB accepts full responsibility for the work and the conduct of the study, had access to the data, and controlled the decision to publish.

## FUNDING INFORMATION

The research leading to the results for CONSIGN was conducted as part of the activities of the EU PE&PV Research Network, which is a public academic partnership coordinated by the Utrecht University (UU), The Netherlands. The project has received support from the EMA under the Framework service contract nr EMA/2018/28/PE and was scientifically coordinated by the University Medical Center Utrecht (UMCU). The content of this document expresses the opinion of the authors and may not be understood or quoted as being made on behalf of or reflecting the position of the EMA or one of its committees or working parties. Electronic healthcare data sources participating in the CONSIGN EHR study were partly funded by the EMA under the above‐mentioned Framework service contract. Each of the other participating sites in this meta‐analysis has its own funding to collect the data and generate the evidence. The Canadian Mother–Child (CAMCCO) Active Surveillance Initiative is a pan‐Canadian program on drug safety and efficacy in pregnancy funded by the Canadian Institutes of Health Research (CIHR), and the Canada Foundation for Innovation (CFI), that is scientifically coordinated by CHU Sainte‐Justine in Montreal, Quebec, Canada. The Sentinel System is a U.S. government initiative managed and funded by the U.S. Food and Drug Administration (FDA) and was scientifically coordinated by Harvard Pilgrim Health Care Institute. This project was supported by Task Order 75F40122F19005 and 75F40119F19001 under Master Agreements HHSF223201400030I and 75F40119D10037, from FDA. The FDA approved the study protocol including statistical analysis plan and reviewed and approved this manuscript. Coauthors from the FDA (JH, LS, WH) participated in the results interpretation and in the preparation and decision to submit the manuscript for publication. The FDA had no role in data collection, management, or analysis.

## CONFLICT OF INTEREST STATEMENT

OdB, EM, EH, HMEN, AB, OS, PK, MUS, AC, JGL, EMJ, MEK, ST, WH, JJHM, LS, RG, OP, BPP, SJ, CLRB, FSS, RL, MAB, GF, AP, KP, CdV, and SJS have no conflicts of interest to declare. CEC, DH, FA, KWMB, and MCJM report participation in research studies funded by pharmaceutical companies, with all funds paid to the institution where they are employed (no personal fees).

## Supporting information


Data S1.

## Data Availability

All information and materials presented in this manuscript are original. The aggregated results from the individual data sources that support the findings of this study are available on request from the corresponding author. The individual level data in each data source are not publicly available due to privacy, governance or ethical restrictions.
