# Supplementary material for: Adverse outcomes among pregnant women with COVID‐19 according to hospitalization status: A prospective individual participant data meta‐analysis in Europe and North America
Source: Int J Gynaecol Obstet. 2025 Dec 15;173(3):1197–206. doi: 10.1002/ijgo.70694 (PMC13173624; doi:10.1002/ijgo.70694)
Supplement: Supplementary file 1 — Data S1. [file IJGO-173-1197-s001.zip › Supplementary information V8_IJGO.docx]

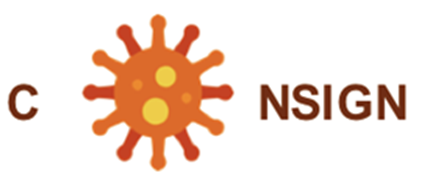


**Covid-19 infectiOn aNd medicineS In pregGNancy**

**SUPPLEMENTARY INFORMATION**

**Adverse outcomes among pregnant women with COVID-19 according to hospitalization status: a prospective individual participant data meta-analysis in Europe and North America**

Odette de Bruin^1,2^, Emeline Maisonneuve^3,4,5^, Eimir Hurley^6^, Hedvig M.E. Nordeng^6,7^, Anick Bérard^8,9^, Odile Sheehy^8,9^, Padma Kaul^10^, Mayura U. Shinde^11^, Austin Cosgrove^12^, Jennifer G. Lyons^11^, Elizabeth Messenger-Jones^12^, Maria E. Kempner^12^, Sengwee D. Toh^11^, Wei Hua^13^, José J. Hernández-Muñoz^13^, Leyla Sahin^14^, Carolyn E. Cesta^15^, David Hägg^15^, Rosa Gini^16^, Olga Paoletti^16^, Beatriz Poblador-Plou^17,18^, Sue Jordan^19^, Clara L. Rodríguez-Bernal^18,20^, Francisco Sánchez-Sáez^18,20^, Régis Lassalle^21^, Marie-Agnès Bernard^21^, Fariba Ahmadizar^1^, Guillaume Favre^5^, Alice Panchaud^3,5,22^, Kitty W.M. Bloemenkamp^2^, Kelly Plueschke^23^, Corinne de Vries^23^, Satu J. Siiskonen^24^, and Miriam C.J.M. Sturkenboom^1^, on behalf of the CONSIGN collaboration group^*^

^1^Department of Data Science & Biostatistics, Julius Global Health, University Medical Center Utrecht (UMCU), Utrecht, the Netherlands

^2^Department of Obstetrics, Division Woman and Baby, Wilhelmina Children’s Hospital, University Medical Center Utrecht (UMCU), Utrecht, the Netherlands

^3^Institute of Primary Health Care (BIHAM), University of Bern, Bern, Switzerland

^4^Graduate School for Health Sciences (GHS), University of Bern, Bern, Switzerland

^5^Materno-Fetal and Obstetrics Research Unit, Woman-Mother-Child Department, Lausanne University Hospital, Lausanne, Switzerland

^6^Pharmacoepidemiology and Drug Safety Research Group, Department of Pharmacy, University Oslo (UiO), Oslo, Norway

^7^Department of Child Health and Development, Norwegian Institute of Public Health, Oslo, Norway

^8^Faculty of Pharmacy, University of Montreal, Montreal, Quebec, Canada

^9^Centre Hospitalier Universitaire (CHU) de Sainte-Justine, Montreal, Quebec, Canada

^10^University of Alberta, Edmonton, Alberta, Canada

^11^Department of Population Medicine, Harvard Medical School and Harvard Pilgrim Health Care Institute, Boston, Massachusetts, USA

^12^Department of Population Medicine, Harvard Pilgrim Health Care Institute, Boston, Massachusetts, USA

^13^Office of Surveillance and Epidemiology, Center for Drug Evaluation and Research, U.S. Food and Drug Administration, Silver Spring, USA

^14^Office of New Drugs, Center for Drug Evaluation and Research, U.S. Food and Drug Administration, Silver Spring, USA

^15^Department of Medicine Solna, Centre for Pharmacoepidemiology, Karolinska Institutet, Stockholm, Sweden

^16^Tuscan Regional Healthcare Agency, Florence, Italy

^17^EpiChron Research Group, Aragon Health Sciences Institute (IACS), IIS Aragón, Miguel Servet University Hospital, Zaragoza, Spain

^18^Network for Research on Chronicity, Primary Care and Health Promotion (RICAPPS), Research Network on Health Services in Chronic Diseases, Institute of Health Carlos III, Madrid, Spain

^19^Faculty of Medicine, Health and Life Science, Swansea University, Swansea, Wales, UK

^20^Health Services Research and Pharmacoepidemiology Unit, Foundation for the Promotion of Health and Biomedical Research of Valencia Region, Valencia, Spain

^21^Bordeaux PharmacoEpi, INSERM CIC-P1401, Université de Bordeaux, Bordeaux, France

^22^Service of Pharmacy, Lausanne University Hospital and University of Lausanne, Lausanne, Switzerland

^23^European Medicines Agency, Amsterdam, the Netherlands

^24^Division of Pharmacoepidemiology and Clinical Pharmacology, Utrecht Institute for Pharmaceutical Sciences (UIPS), Utrecht University, Utrecht, the Netherlands

^*^Collaborators of CONSIGN are listed in Table S1

**Table of contents**

[**Table S1.** Members of the CONSIGN collaboration group 4](#_Toc191453133)

[**Table S2.** Safety outcomes of interest 6](#_Toc191453134)

[**Table S3.** Covariates of interest 7](#_Toc191453135)

[**Table S4.** Overview of differences in analyses between data sources 8](#_Toc191453136)

[**Table S5.** Description of the data sources included in the meta-analysis 9](#_Toc191453137)

[**Figure S1.** Counts of COVID-19 infection in the pregnancy cohort by data source and calendar time 11](#_Toc191453138)

[**Table S6.** Data source specific methods for determining pregnancy start and end and COVID-19 diagnosis 12](#_Toc191453139)

[**Table S7.** Baseline characteristics of pregnant women with and without COVID-19 13](#_Toc191453140)

[**Table S8.** Prevalence of maternal death and its association with COVID-19 by trimester and hospitalization status 14](#_Toc191453141)

[**Table S9.** Prevalence of gestational diabetes and its association with COVID-19 in first trimester by hospitalization status 15](#_Toc191453142)

[**Figure S2.** Gestational diabetes and its association with COVID-19 infection in first trimester by hospitalization status 16](#_Toc191453143)

[**Table S10.** Prevalence of pre-eclampsia and its association with COVID-19 in first trimester by hospitalization status 17](#_Toc191453144)

[**Figure S3.** Pre-eclamspia and its association with COVID-19 infection in first trimester by hospitalization status 18](#_Toc191453145)

[**Table S11.** Prevalence of caesarean section and its association with COVID-19 by trimester and hospitalization status 19](#_Toc191453146)

[**Figure S4.** Caesarean section and its association with COVID-19 infection in first trimester by hospitalization status 20](#_Toc191453147)

[**Figure S5.** Caesarean section and its association with COVID-19 infection in second trimester by hospitalization status 21](#_Toc191453148)

[**Figure S6.** Caesarean section and its association with COVID-19 infection in third trimester by hospitalization status 22](#_Toc191453149)

[**Table S12.** Prevalence of preterm birth and its association with COVID-19 by trimester and hospitalization status 23](#_Toc191453150)

[**Figure S7.** Preterm birth and its association with COVID-19 infection in first trimester by hospitalization status 24](#_Toc191453151)

[**Figure S8.** Preterm birth and its association with COVID-19 infection in second trimester by hospitalization status 25](#_Toc191453152)

[**Figure S9.** Preterm birth and its association with COVID-19 infection in third trimester by hospitalization status 26](#_Toc191453153)

[**Table S13.** Prevalence of stillbirth and its association with COVID-19 by trimester and hospitalization status 27](#_Toc191453154)

[**Figure S10.** Stillbirth and its association with COVID-19 infection in second trimester among non-hospitalized pregnant women 28](#_Toc191453155)

[**Figure S11.** Stillbirth and its association with COVID-19 infection in third trimester by hospitalization status 29](#_Toc191453156)

[**Table S14.** Prevalence of neonatal death and its association with COVID-19 by trimester and hospitalization status 30](#_Toc191453157)

[**Table S15.** Prevalence of low birth weight and its association with COVID-19 by trimester and hospitalization status 31](#_Toc191453158)

[**Figure S12.** Low birth weight and its association with COVID-19 infection in first trimester by hospitalization status 32](#_Toc191453159)

[**Figure S13.** Low birth weight and its association with COVID-19 infection in second trimester by hospitalization status 33](#_Toc191453160)

[**Figure S14.** Low birth weight and its association with COVID-19 infection in third trimester by hospitalization status 34](#_Toc191453161)

[**Table S16.** Prevalence of small for gestational age and its association with COVID-19 by trimester and hospitalization status 35](#_Toc191453162)

[**Figure S15.** Small for gestational age and its association with COVID-19 infection in first trimester among non-hospitalized pregnant women 36](#_Toc191453163)

[**Figure S16.** Small for gestational age and its association with COVID-19 infection in second trimester by hospitalization status 37](#_Toc191453164)

[**Figure S17.** Small for gestational age and its association with COVID-19 infection in third trimester by hospitalization status 38](#_Toc191453165)

[**Table S17.** Prevalence of major congenital abnormalities and its association with COVID-19 in first trimester by hospitalization status 39](#_Toc191453166)

[**Figure S18.** Major congenital abnormalties and its association with COVID-19 infection in first trimester by hospitalization status 40](#_Toc191453167)

# **Table S1.** Members of the CONSIGN collaboration group

| **Organization** | **Name** | **Affiliation** | **ORCID ID** |
| --- | --- | --- | --- |
| University of Oslo (CONSIGN WP1) | Benjamin P. Geisler | Pharmacoepidemiology and Drug Safety Research Group, Department of Pharmacy, University Oslo (UiO), Oslo, Norway | 0000-0003-1704-6067 |
| CAMCCO | Mark Walker | University of Ottawa, Ottawa, Ontario, Canada | 0000-0001-8974-4548 |
| CAMCCO | Steven Hawken | University of Ottawa, Ottawa, Ontario, Canada | 0000-0002-3341-9022 |
| CAMCCO | Sasha Bernatsky | Department of Epidemiology, Biostatistics and Occupational Health, McGill University, Montreal, Quebec, Canada | 0000-0002-9515-2802 |
| CAMCCO | Sherif Eltonsy | University of Manitoba, Winnipeg, Manitoba, Canada | 0000-0002-0520-5406 |
| Sentinel System | Emma Hoffman | Department of Population Medicine, Harvard Pilgrim Health Care Institute, Boston, Massachusetts, USA | N.A. |
| Sentinel System | Andrew B. Petrone | Department of Population Medicine, Harvard Pilgrim Health Care Institute, Boston, Massachusetts, USA | 0000-0001-8413-6236 |
| Sentinel System | Jolene Mosley | Department of Population Medicine, Harvard Pilgrim Health Care Institute, Boston, Massachusetts, USA | N.A. |
| Sentinel System | Jenice Ko | Department of Population Medicine, Harvard Pilgrim Health Care Institute, Boston, Massachusetts, USA | N.A. |
| ARS Toscana | Claudia Bartolini | Tuscan Regional Healthcare Agency, Florence, Italy | 0000-0001-8630-4598 |
| ARS Toscana | Giuseppe Roberto | Tuscan Regional Healthcare Agency, Florence, Italy | N.A. |
| ARS Toscana | Giorgio Limoncella | Tuscan Regional Healthcare Agency, Florence, Italy | N.A. |
| ARS Toscana | Anna Girardi | Tuscan Regional Healthcare Agency, Florence, Italy | N.A. |
| ARS Toscana | Giulia Hyeraci | Tuscan Regional Healthcare Agency, Florence, Italy | 0000-0002-6536-2083 |
| IACS | Antonio Gimeno-Miguel | EpiChron Research Group, Aragon Health Sciences Institute (IACS), IIS Aragón, Miguel Servet University Hospital, Zaragoza, Spain | 0000-0002-5440-1710 |
| IACS | Jonás Carmona-Pírez | EpiChron Research Group, Aragon Health Sciences Institute (IACS), IIS Aragón, Miguel Servet University Hospital, Zaragoza, Spain | 0000-0002-6268-8803 |
| IACS | Antonio Poncel-Falcó | EpiChron Research Group, Aragon Health Sciences Institute (IACS), IIS Aragón, Miguel Servet University Hospital, Zaragoza, Spain | N.A. |
| IACS | Aida Moreno-Juste | EpiChron Research Group, Aragon Health Sciences Institute (IACS), IIS Aragón, Miguel Servet University Hospital, Zaragoza, Spain | 0000-0002-8819-3278 |
| IACS | Alexandra Prados-Torres | EpiChron Research Group, Aragon Health Sciences Institute (IACS), IIS Aragón, Miguel Servet University Hospital, Zaragoza, Spain | 0000-0002-5704-6056 |
| SWANSEA | Daniel Thayer | Faculty of Medicine, Health and Life Science, Swansea University, Wales, UK | N.A. |
| SWANSEA | Ian Farr | Faculty of Medicine, Health and Life Science, Swansea University, Wales, UK | N.A. |
| SWANSEA | Saira Ahmed | Faculty of Medicine, Health and Life Science, Swansea University, Wales, UK | N.A. |
| SWANSEA | Ieuan Scanlon | Faculty of Medicine, Health and Life Science, Swansea University, Wales, UK | N.A. |

**Table S1 continued.** Members of the CONSIGN collaboration group

| **Organization** | **Name** | **Affiliation** | **ORCID ID** |
| --- | --- | --- | --- |
| FISABIO-HSRU | Gabriel Sanfélix-Gimeno | Health Services Research and Pharmacoepidemiology Unit, Foundation for the Promotion of Health and Biomedical Research of Valencia Region, Valencia, Spain | 0000-0001-7098-4576 |
| FISABIO-HSRU | Isabel Hurtado | Health Services Research and Pharmacoepidemiology Unit, Foundation for the Promotion of Health and Biomedical Research of Valencia Region, Valencia, Spain | 0000-0002-8475-8112 |
| FISABIO-HSRU | Anibal Garcia-Sempere | Health Services Research and Pharmacoepidemiology Unit, Foundation for the Promotion of Health and Biomedical Research of Valencia Region, Valencia, Spain | N.A. |
| FISABIO-HSRU | Salvador Peiro | Health Services Research and Pharmacoepidemiology Unit, Foundation for the Promotion of Health and Biomedical Research of Valencia Region, Valencia, Spain | 0000-0002-3902-569X |
| BPE | Jérémy Jové | Bordeaux PharmacoEpi, INSERM CIC-P1401, Université de Bordeaux, Bordeaux, France | N.A. |
| BPE | Dunia Sakr | Bordeaux PharmacoEpi, INSERM CIC-P1401, Université de Bordeaux, Bordeaux, France | N.A. |
| BPE | Cécile Droz-Perroteau | Bordeaux PharmacoEpi, INSERM CIC-P1401, Université de Bordeaux, Bordeaux, France | 0000-0002-7697-1167 |
| UMC Utrecht | Ema Alsina | Department of Data Science & Biostatistics, Julius Global Health, University Medical Center Utrecht (UMCU), Utrecht, the Netherlands | N.A. |
| COVI-PREG  (CONSIGN WP2) | David Baud | Materno-Fetal and Obstetrics Research Unit, Woman-Mother-Child Department, Lausanne University Hospital, Lausanne, Switzerland | N.A. |
| INOSS  (CONSIGN WP3) | Hilde M. Engjom | Department for Health Promotion and Department for Health Registry Research and Development, Norwegian Institute of Public Health, Bergen, Norway | 0000-0003-1582-4283 |
| Vall d’Hebron & UMC Utrecht | Judit Riera-Arnau | Department of Clinical Pharmacology, Vall d'Hebron Hospital Universitari, Vall Hebron Institut de Recerca Barcelona, Spain & Department of Data Science & Biostatistics, Julius Global Health, University Medical Center Utrecht (UMCU), Utrecht, the Netherlands | 0000-0001-7591-0218 |
| Vall d’Hebron | Mònica Sabaté Gallego | Department of Clinical Pharmacology, Vall d'Hebron Hospital Universitari, Vall Hebron Institut de Recerca Barcelona, Spain | 0000-0001-6206-1085 |
| Vall d’Hebron | Elena Ballarín Alins | Department of Clinical Pharmacology, Vall d'Hebron Hospital Universitari, Vall Hebron Institut de Recerca Barcelona, Spain | 0000-0001-9786-6617 |
| Vall d’Hebron | Cristina Aguilera Martin | Department of Clinical Pharmacology, Vall d'Hebron Hospital Universitari, Vall Hebron Institut de Recerca Barcelona, Spain | 0000-0002-7985-7327 |
| Health Canada | Melissa Kampman | Data Analytics and Real-world Evidence Division, Health Products and Food Branch, Health Canada, Ottawa, Ontario, Canada | N.A. |
| Health Canada | Celline Brasil | Data Analytics and Real-world Evidence Division, Health Products and Food Branch, Health Canada, Ottawa, Ontario, Canada | 0000-0002-6324-4702 |

N.A. = not available.

# **Table S2.** Safety outcomes of interest

| **Outcomes** | **Clinical Definition** | **Operationalization of outcomes** |
| --- | --- | --- |
| **Maternal outcomes** | | |
| Maternal death^[[1]](#footnote-2)^ | The death of a woman while pregnant or within 42 days of termination of pregnancy, irrespective of the duration and site of the pregnancy, from any cause related to or aggravated by the pregnancy or its management but not from accidental or incidental causes. | Maternal date of death during pregnancy or in the six weeks after resolution of the pregnancy OR diagnostic codes for maternal death |
| Preeclampsia^[[2]](#footnote-3)^ | Preeclampsia is a clinical syndrome characterized by pregnancy ≥ 20 weeks AND new onset hypertension (systolic blood pressure ≥ 140 mmHg and/or diastolic blood pressure ≥ 90 mmHg) sustained on two measurements over a minimum of 1h AND new onset proteinuria. | Recording of ‘pre-eclampsia’ in registry data OR diagnostic codes |
| Gestational diabetes mellitus (GDM)^[[3]](#footnote-4)^ | Gestational diabetes mellitus is a clinical syndrome characterized by the absence of pre-gestational diabetes diagnosis defined by previous diagnosis of diabetes while not pregnant OR first trimester hemoglobin A1c level of ≥ 6.5% OR first trimester fasting blood glucose >126 mg/dL or >6.93 mmol/L AND identification of sustained hyperglycemia during pregnancy not due to other known causes. | Recording of ‘gestational diabetes’ in registry data OR diagnostic codes |
| **Pregnancy outcomes** | | |
| Caesarean section | Delivery of fetus via abdominal incision (laparotomy) and then uterine incision (hysterotomy). | Recording of ‘caesarean section’ in registry data OR diagnostic/procedure codes |
| Preterm birth^[[4]](#footnote-5)^ | Any birth before 37 completed weeks of gestation, or fewer than 259 days since the first day of the individual’s last menstrual period (LMP). This is further subdivided into: extremely preterm (<28 weeks), very preterm (28–<32 weeks), moderate or late preterm (32–<37 weeks). | Duration of pregnancy < 37 weeks OR diagnostic codes |
| Stillbirth^[[5]](#footnote-6)^ | Fetal death occurring before birth after a selected predefined duration of gestation. There are several definitions for the minimal gestational age: WHO/ICD/EMA = 22 weeks, ACOG/CDC = 20 weeks, UK = 24 weeks. The death of the fetus could have occurred before onset of labor (antepartum) or at the delivery (intrapartum). WHO/ICD defines stillbirth as the death of a fetus that has reached a birth weight of 500 g, or if unavailable, 22 weeks gestation or crown-to-heel length of 25 cm. | Recording of ‘stillbirth’ in registry data OR diagnostic codes |
| **Neonatal outcomes** | | |
| Neonatal death^[[6]](#footnote-7)^ | Death of a live-born baby within the first 28 days of life. Early neonatal mortality refers to the death of a live-born baby within the first 7 days of life, while late neonatal mortality refers to death after 7 days until before 28 days. | Based on date of death of neonate within 28 days of date of birth OR diagnostic codes for neonatal death |
| Low birth weight (LBW)^[[7]](#footnote-8)^ | Body weight of the newborn at birth of less than 2500 grams (up to and including 2499 g) regardless of their gestational age. | Recording of child’s weight less than 2500 gram in registry data OR diagnostic codes |
| Small-for-gestational age (SGA)^[[8]](#footnote-9)^ | Newborns who have a birth weight lower than expected for their gestational age, based on population-based or standard growth charts. SGA babies may have experienced intrauterine growth restriction, which means they did not grow adequately during pregnancy. | Raw z-score based on baby's weight, gestational age and sex, in registry data OR diagnostic codes |
| Major congenital anomalies^[[9]](#footnote-10)^ | Congenital anomalies are conditions of prenatal origin that are present at birth. Anomalies which affect an infant’s life expectancy, health status, physical or social functioning may be described as “major” anomalies e.g. cleft lip and spina bifida. For all levels of diagnostic certainty counts that a major congenital anomaly is a structural or functional defect with the following three characteristics 1) Of prenatal origin, 2) Present at the time of live birth or fetal demise, or in utero, 3) Affecting the health, survival, or physical or cognitive functioning of the individual. | Recording of major congenital anomalies in registry data OR diagnostic codes within the first 3 months of life |

Abbreviations: ACOG = American College of Obstetricians and Gynecologists; CDC = Centers for Disease Control and Prevention; EMA= European Medicines Agency; ICD = International Classification of Diseases; WHO = World Health Organization

# **Table S3.** Covariates of interest

| **Covariate** | **Definition** | **Identification of covariate** |
| --- | --- | --- |
| Age of mother | Categorized into three groups: 12-24 years of age, 25-39 years of age, 40-55 years of age in CONSIGN EHR study and Sentinel. In CAMCCO, the age groups are 15-24 years of age, 25-39 years of age, and 40-45 years of age. | Date of birth |
| Trimester of pregnancy | For CONSIGN EHR, the ACOG definition of timing in pregnancy is used:   1. Trimester 1: from LMP to day 97 after LMP; or end of pregnancy, whichever earlier 2. Trimester 2: from day 98 after LMP to day 195 after LMP; or end of pregnancy, whichever earlier 3. Trimester 3: from day 196 after LMP onwards until end of pregnancy   For Sentinel:   1. First trimester: days 0 to 90 of gestation (13 weeks) 2. Second trimester: days 91 to 180 (13+1 to 25+5 weeks) 3. Third trimester: days 181 (≥25+6 weeks through the day of the hospital admission for live-birth delivery.   For CAMCCO:   1. 1^st^ trimester: 0-98 days (> 0 to ≥14 weeks gestation) 2. 2^nd^ trimester: 99-182 days (>14 to ≥26 weeks’ gestation) 3. 3^rd^ trimester: ≥183 days (≥26+1 weeks’ gestation to end of the pregnancy) | Pregnancy algorithm |
| Calendar month of COVID-19 diagnosis | December 2019 till [according to each data source] | Date of diagnosis/positive test |
| Hospitalization status | In CONSIGN data sources, non-hospitalized women had a recording of a positive COVID-19 test or diagnosis, with no subsequent hospital admission with a recording (primary or secondary) of COVID-19 (within a 4-week period). Hospitalized women had any recording of ‘COVID-19 positive test’, or ‘COVID-19 complication’ in any of the diagnostic fields in hospital records, not just the principal diagnosis. However, if the COVID-19 test positive test was two days of delivery date/hospitalization for obstetric reasons, and no codes of severe symptoms (pneumonia, respiratory aid/use of ventilator) were found subsequently, these women were excluded from the hospitalized COVID-19 group. This was not conducted in Sentinel, where hospitalized COVID-19 was defined as hospitalized patients with COVID-19 complications, ICU admission, ventilation, or death; and non-hospitalized COVID-19 as diagnosis of COVID-19 or positive SARS-COV-2 test. The Sentinel System ensured temporality to exclude any pregnancies that had evidence of the maternal outcome prior to date of COVID-19. |  |
| At-risk medical conditions for severe COVID-19 | At risk conditions for severe COVID-19 were divided into following subcategories:   - Cardiovascular disease/serious heart conditions include heart failure, coronary artery disease, cardiac myopathies - Hypertension - Sickle cell disease - Chronic lung disease including COPD, cystic fibrosis, severe asthma, interstitial lung disease, pulmonary hypertension, bronchiectasis - Type 1 & 2 Diabetes - Obesity diagnosis or having a BMI ≥30 kg/m2 - Chronic kidney disease - Chronic liver disease diagnosis (cirrhosis, non-alcoholic fatty liver disease, alcoholic liver disease, autoimmune hepatitis) - HIV - Common rheumatic diseases - Immunosuppression & solid organ transplants - Cancer - Mental health disease (depression, dementia, and schizophrenia spectrum disorders) | Recording of the at-risk medical conditions for severe COVID-19 OR diagnostic codes AND medicines proxies |
| Risk conditions for obstetric complications | Prior maternal history of gestational diabetes or pre-eclampsia and prior history of stillbirth or late miscarriage, small for gestational age (SGA) child or fetal growth restriction (FGR), or child with congenital anomaly. | Recording of risk conditions for obstetric complications OR diagnostic codes |

Abbreviations: ACOG = American College of Obstetricians and Gynecologists; BMI = body mass index; CAMCCO = Canadian Mother-Child Cohort; EHR = electronic health record; LMP = last menstrual period; PCR = polymerase chain reaction.

# **Table S4.** Overview of differences in analyses between data sources

|  | **CONSIGN EHR** | **CAMMCO** | **Sentinel System** |
| --- | --- | --- | --- |
| **Matching** | 1:3 | No matching | 1:1 |
| **Model** | Modified Poisson model for live-birth outcomes  Cox proportional hazard model for non-live birth outcomes | Log-binomial regression models and the link function logit for all outcomes | Modified Poisson model for live-birth outcomes |

# **Table S5.** Description of the data sources included in the meta-analysis

| **Data access provider**  **(data source)** | **Country**  **(area)** | **Estimated births per year** | **Type of data source** | **Medical birth registry** | **Diagnosis** | **Data availability** |
| --- | --- | --- | --- | --- | --- | --- |
| **CONSIGN EHR study** | | | | | | |
| ARS Toscana  (ARS database) | Italy  (Tuscany) | 25 000 | Record linkage | Yes | In-hospital, emergency room | Mar 2020 – Dec 2021 |
| BPE  (SNDS) | France  (national) | 700 000 | Health insurance | No | In-hospital | Mar 2020 – Dec 2020 |
| FISABIO-HSRU  (VID) | Spain  (Valencia) | 32 000 | Record linkage | Yes | GP, In-hospital, Outpatient Specialists | Mar 2020 – Dec 2021 |
| IACS  (PRECOVID study aNd EpiChron Cohort) | Spain  (Aragon) | 10 000 | Record linkage | Yes | GP, In-hospital | Mar 2020 – Dec 2021 |
| UiO  (Linked national registries) | Norway  (national) | 60 000 | Record linkage | Yes | GP, In-hospital | Mar 2020 – Dec 2021 |
| Karolinska Institutet  (Linked national registers) | Sweden  (national) | 100 000 | Record linkage | Yes | In-hospital, Outpatient Specialists | Mar 2020 – Dec 2020 |
| **Total estimated births Europe:** | | **960 000 births/year** | | | | |
| **CAMCCO** | | | | | | |
| Alberta  (Linked databases) | Canada  (Alberta) | 49 000 | Record linkage | Yes | GP, In-hospital, Outpatient Specialists, COVID-19 database | Mar 2020 - Aug 2021 |
| Manitoba  (Linked databases) | Canada  (Manitoba) | 16 000 | Record linkage | Yes | GP, In-hospital, Outpatient Specialists, COVID-19 database | Mar 2020 - Feb 2021 |
| Ontario  (Linked databases) | Canada  (Ontario) | 136 000 | Record linkage | Yes | GP, In-hospital, Outpatient Specialists, COVID-19 database | Mar 2020 - June 2021 |
| **Total estimated births in Canada:** | | **201 000 births/year** | | | | |
| **Sentinel System** | | | | | | |
| Sentinel System  (Sentinel Distributed Database) | US  (national and regional coverage in Colorado, Oregon, Minnesota, and Washington States) | 485 000 | Health insurance | No | In-Hospital, Outpatient  Specialists | Jan 2020 - Dec 2022 |
| **Total estimated births in US:** | | **485 000 births/year** | | | | |
| **Total estimated births:** | | **1 685 000 births/year** | | | | |

Abbreviations: ARS = Agenzia Regionale di Sanita’ della Toscana; BPE = Bordeaux PharmacoEpi platform; CAMCCO = Canadian Mother-Child Cohort; EHR = electronic health record; FISABIO-HSRU = Foundation for the Promotion of Health and Biomedical Research of Valencia Region - Health Services Research Unit; GP: general practitioner, primary care; IACS = Instituto Aragones de Ciencias de la Salud; SNDS = Système National des Données de Santé; UiO = University of Oslo; VID = Valencia Integrated Database.


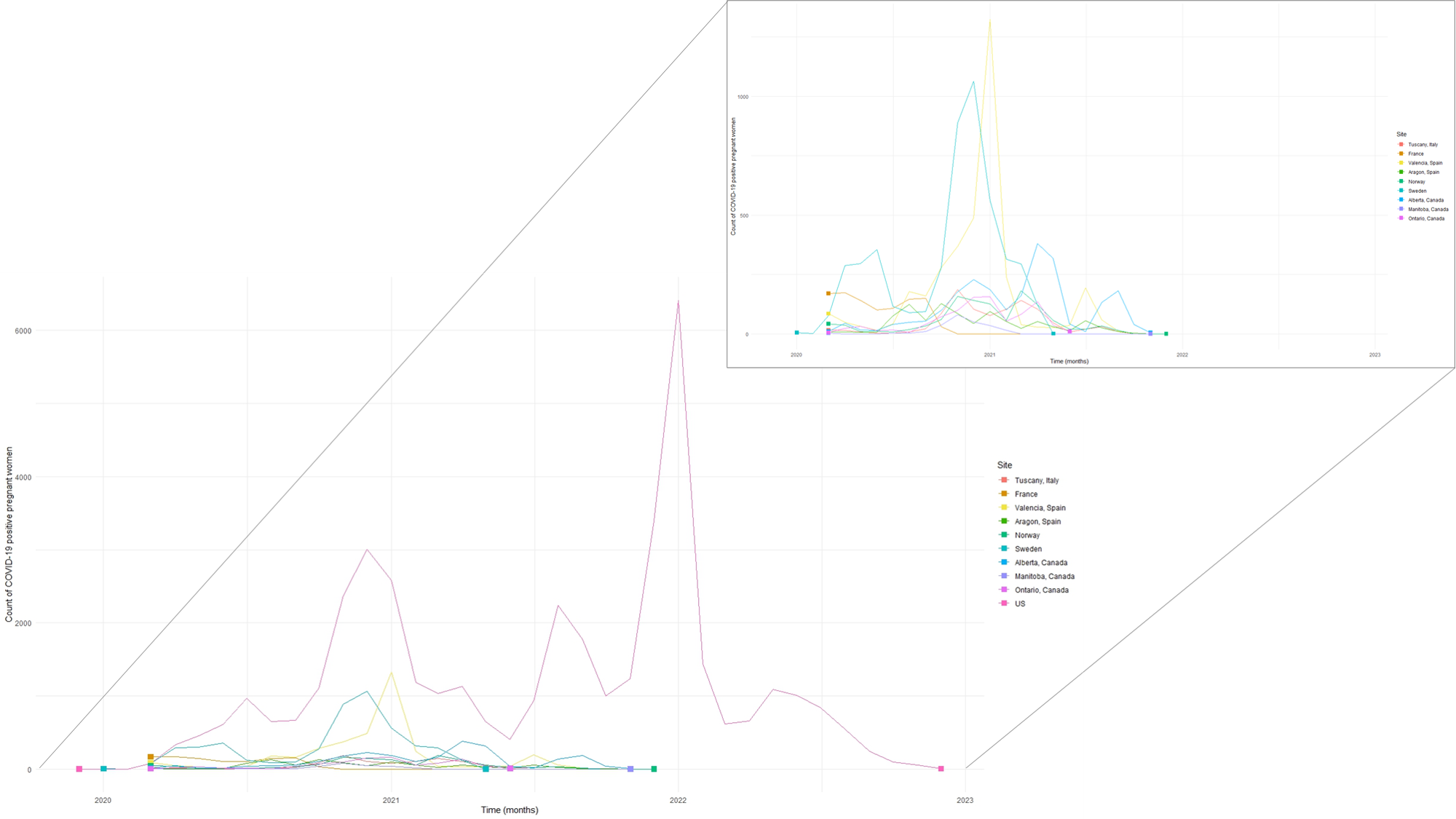


# **Figure S1.** Counts of COVID-19 infection in the pregnancy cohort by data source and calendar time

# **Table S6.** Data source specific methods for determining pregnancy start and end and COVID-19 diagnosis

| **DAP**  **(data source)** | **Pregnancy start** | **Pregnancy end** | **COVID-19 diagnosis** |
| --- | --- | --- | --- |
| ARS Toscana (ARS database) | Date of record minus gestational age OR estimated on diagnostic code or period of pregnancy when a procedure is first expected | Date of record OR  estimated from due  date based on estimation of start date | Registry of positive COVID-19 tests (official surveillance system) |
| BPE  (SNDS) | Date estimated from pregnancy algorithm which includes LMP and gestational age | Date of delivery | Inpatient data (PMSI) with ICD10 codes for COVID-19 diagnoses; no laboratory positive test result available. |
| FISABIO-HSRU  (VID) | Date of delivery/ end of pregnancy minus gestational age (based on ultrasound/LMP) (95% pregnancies) OR Date of delivery minus 40 weeks when new-borns weight is 2500 gr or above and through a linear model when weight <2500 gr | Date of delivery or  abortion | All PCR or antigen test results are recorded in RedMIVA (Microbiological Surveillance Network of the Valencian Community) |
| IACS  (PRECOVID study aNd EpiChron Cohort) | Date of the LMP | Date of the pregnancy  delivery or abortion recorded in primary care or hospital discharge | Registry developed for monitoring the evolution of COVID-19 disease in the region, includes all PCR or antigen test results |
| UiO  (Linked national registries) | Date of delivery minus gestational length in days based on ultrasound or LMP | Date of delivery (all pregnancies > gestational week 12, live or non-live) | Laboratory confirmed positive test recorded in MSIS (Norwegian surveillance system for communicable  diseases) |
| Karolinska Institutet  (Linked national registries) | Date of delivery minus  gestational length in days  based on ultrasound or LMP | Date of delivery | All positive PCR test results registered in SmiNet (The Infectious Disease Register) and ICD-10 diagnosis for COVID-19 (National Patient register) |
| Alberta  (Linked databases) | First day of LMP (for deliveries) using gestational age reported in the delivery hospitalization chart summary AND algorithm to estimate first day of LMP (for spontaneous and planned abortions) | Clinically detected  spontaneous or  induced/planned abortion or  delivery | PCR test |
| Manitoba  (Linked databases) | First day of LMP (for deliveries) using gestational age reported in the delivery hospitalization chart summary AND algorithm to estimate first day of LMP (for spontaneous and planned abortions) | Clinically detected  spontaneous or  induced/planned abortion; or  delivery | PCR test |
| Ontario  (Linked databases) | First day of LMP (for deliveries) using gestational age reported in the delivery hospitalization chart summary AND algorithm to estimate first day of LMP (for spontaneous and planned abortions) | Clinically detected  spontaneous or  induced/planned abortion; or  delivery | PCR test |
| Sentinel System  (Sentinel Distributed Database) | Estimated based on date of live-birth delivery and gestational age codes surrounding delivery date | ICD-10 diagnosis code  indicating live-birth  delivery | ICD-10 diagnosis code for  COVID-19 and/or positive  COVID-19 NAAT test |

Abbreviations: ARS = Agenzia Regionale di Sanita’ della Toscana; BPE = Bordeaux PharmacoEpi platform; CAMCCO = Canadian Mother-Child Cohort; EHR = electronic health record; FISABIO-HSRU = Foundation for the Promotion of Health and Biomedical Research of Valencia Region - Health Services Research Unit; IACS = Instituto Aragones de Ciencias de la Salud; LMP = last menstrual period; NAAT = Nucleic Acid Amplification Tests; PCR = polymerase chain reaction; SNDS = Système National des Données de Santé; SWANSEA = Swansea University; UiO = University of Oslo; VID = Valencia Integrated Database.

# **Table S7.** Baseline characteristics of pregnant women with and without COVID-19

| **Study site** | **Age** | | **Co-morbidities^1^** | | **Obstetric risk^2^** | |
| --- | --- | --- | --- | --- | --- | --- |
|  | **Pregnant with**  **COVID-19** | **Pregnant without**  **COVID-19** | **Pregnant with**  **COVID-19** | **Pregnant without**  **COVID-19** | **Pregnant with**  **COVID-19** | **Pregnant without**  **COVID-19** |
| Tuscany, Italy | 12-24 years: 11.1%  25-39 years: 81.6%  40-55 years: 7.3% | 12-24 years: 11.1%  25-39 years: 81.6%  40-55 years: 7.3% | Any: 20.8% Cardiovascular: 4.2%  Chronic lung: 4.6%  Severe obesity: 0.2% | Any: 22.1% Cardiovascular: 4.2%  Chronic lung: 5.7%  Severe obesity: 0.2% | Any: 8.4% Adverse pregnancy outcomes: 7.0% | Any: 6.4% Adverse pregnancy outcomes: 5.2% |
| Valencia, Spain | 12-24 years: 13.2%  25-39 years: 76.9%  40-55 years: 9.9% | 12-24 years: 13.2%  25-39 years: 76.9%  40-55 years: 9.9% | Any: 23.6% Cardiovascular: 5.9%  Chronic lung: 9.9%  Severe obesity: 1.7% | Any: 21.5% Cardiovascular: 4.4%  Chronic lung: 9.0%  Severe obesity: 1.4% | Any: 12.0% Adverse pregnancy outcomes: 9.6% | Any: 7.1% Adverse pregnancy outcomes: 5.5% |
| Aragon, Spain | 12-24 years: 13.9%  25-39 years: 78.3%  40-55 years: 7.8% | 12-24 years: 13.9%  25-39 years: 78.3%  40-55 years: 7.8% | Any: 24.6% Cardiovascular: 4.4%  Chronic lung: 7.5%  Severe obesity: 0.4% | Any: 24.4% Cardiovascular: 3.3%  Chronic lung: 8.3%  Severe obesity: 0.6% | Any: 10.4% Adverse pregnancy outcomes: 8.5% | Any: 9.1% Adverse pregnancy outcomes: 7.0% |
| Norway | 12-24 years: 10.6%  25-39 years: 86.2%  40-55 years: 3.2% | 12-24 years: 10.6%  25-39 years: 86.2%  40-55 years: 3.2% | Any: 19.4% Cardiovascular: 3.6%  Chronic lung: 4.5%  Severe obesity: 1.7% | Any: 21.5% Cardiovascular: 2.7%  Chronic lung: 5.8%  Severe obesity: 1.3% | Any: 2.0% Adverse pregnancy outcomes: 0.4% | Any: 1.7% Adverse pregnancy outcomes: 0.3% |
| Sweden | 12-24 years: 6.2%  25-39 years: 88.5%  40-55 years: 5.3% | 12-24 years: 6.2%  25-39 years: 88.5%  40-55 years: 5.3% | Any: 6.2% Cardiovascular: 0.4%  Chronic lung: 0.8%  Severe obesity: 3.7% | Any: 5.5% Cardiovascular: 0.6%  Chronic lung: 0.7%  Severe obesity: 3.0% | Any: N.A. Adverse pregnancy outcomes: N.A. | Any: N.A. Adverse pregnancy outcomes: N.A. |
| Alberta, Canada | 15-24 years: 15.0%  25-39 years: 81.7%  40-45 years: 3.3% | 12-24 years: 13.7%  25-39 years: 82.9%  40-55 years: 3.5% | Any: 41.9% Cardiovascular: 2.5%  Respiratory: 7.6%  Severe obesity: 1.8% | Any: 43.0% Cardiovascular: 2.7%  Respiratory: 9.0%  Severe obesity: 1.4% | Any: N.A. Adverse reproductive history outcomes: 5.4% | Any: N.A. Adverse reproductive history outcomes: 5.2% |
| Manitoba, Canada | 15-24 years: 27.3%  25-39 years: 68.9%  40-45 years: 3.8% | 12-24 years: 22.5%  25-39 years: 74.2%  40-55 years: 3.4% | Any: 38.7% Cardiovascular: <2.6%  Respiratory: 7.2%  Severe obesity: 3.4% | Any: 42.6% Cardiovascular: 1.4%  Respiratory: 9.2%  Severe obesity: 2.0% | Any: N.A. Adverse reproductive history outcomes: 4.3% | Any: N.A. Adverse reproductive history outcomes: 2.7% |
| Ontario, Canada | 15-24 years: 34.7%  25-39 years: 61.7%  40-45 years: 3.5% | 12-24 years: 34.8%  25-39 years: 61.2%  40-55 years: 4.0% | Any: 28.9% Cardiovascular: 0%  Respiratory: 2.6%  Severe obesity: 1.2% | Any: 33.8% Cardiovascular: 0.03%  Respiratory: 2.7%  Severe obesity: 1.6% | Any: N.A. Adverse reproductive history outcomes: 2.8% | Any: N.A. Adverse reproductive history outcomes: 3.5% |
| U.S. | 12-24 years: 16.7%  25-39 years: 79.9%  40-55 years: 3.4% | 12-24 years: 16.7%  25-39 years: 79.9%  40-55 years: 3.4% | Any: N.A. Cardiovascular: 0.9%  Chronic lung: 6.3%  Severe obesity: 15.4% | Any: N.A. Cardiovascular: 0.8%  Chronic lung: 6.2%  Severe obesity: 15.4% | Any: N.A. Adverse pregnancy outcomes: 5.1% | Any: N.A. Adverse pregnancy outcomes: 5.0% |

^1^Any co-morbidities included: cancer, cardiovascular disease, chronic kidney disease, chronic liver disease, chronic lung disease, common rheumatic disease, diabetes, HIV, hypertension, mental disorders, severe obesity, sickle cell disease, and use of immunosuppressants.^2^Any obstetric risk included: prior history of the following: gestational diabetes, gestational hypertension, pre-eclampsia, HELLP and adverse pregnancy outcomes. Adverse pregnancy outcomes included: stillbirth, spontaneous abortion, SGA, FGR, and major congenital abnormalities. N.A. = not available.

# **Table S8.** Prevalence of maternal death and its association with COVID-19 by trimester and hospitalization status

| Site | PREGNANT WOMEN WITH COVID-19 | | | | | | PREGNANT WOMEN WITHOUT COVID-19 | | |
| --- | --- | --- | --- | --- | --- | --- | --- | --- | --- |
|  | **Non-hospitalized cases** | | | **Hospitalized cases** | | |  |  |  |
|  | *Total N* | *Prevalence %* | *Adjusted RR (95% CI)* | *Total N* | *Prevalence %* | *Adjusted RR (95% CI)* | *Total N* | *Prevalence %* | *Adjusted RR (95% CI)* |
| COVID-19 diagnosis in trimester 1 |  |  |  |  |  |  |  |  |  |
| Tuscany, Italy | 177 | 0% | NA | <10 | 0% | NA | 603 | 0% | reference |
| National, France | 0 | NA | NA | <10 | 0% | NA | 18 | 0% | reference |
| Valencia, Spain | 1231 | 0% | NA | 13 | 0% | NA | 3804 | 0% | reference |
| Aragon, Spain | 176 | 0% | NA | <10 | 0% | NA | 552 | 0% | reference |
| National, Norway | 226 | 0% | NA | 18 | 0% | NA | 732 | 0% | reference |
| National, Sweden | 238 | 0% | NA | 26 | 0% | NA | 797 | 0% | reference |
| Alberta, Canada | 571 | 0% | NA | <10 | 0% | NA | 63 764 | <10 cases | reference |
| Manitoba, Canada | 19 | 0% | NA | 0 | NA | NA | 14 365 | <10 cases | reference |
| Ontario, Canada | 270 | 0% | NA | 0 | NA | NA | 59 391 | <10 cases | reference |
| National, US | 15 774 | <10 cases | 0.67 (0.32 – 1.40) | 67 | 0% | NA | 15 841 | <10 cases | reference |
| COVID-19 diagnosis in trimester 2 |  |  |  |  |  |  |  |  |  |
| Tuscany, Italy | 257 | 0% | NA | 17 | 0% | NA | 822 | 0% | reference |
| National, France | 0 | NA | NA | 85 | 0% | NA | 255 | 0% | reference |
| Valencia, Spain | 1032 | 0% | NA | 12 | 0% | NA | 3141 | 0% | reference |
| Aragon, Spain | 250 | 0% | NA | <10 | 0% | NA | 777 | 0% | reference |
| National, Norway | 369 | 0% | NA | 48 | 0% | NA | 1251 | 0% | reference |
| National, Sweden | 1256 | 0% | NA | 223 | <10 cases | NA | 4515 | 0% | reference |
| Alberta, Canada | 706 | <10 cases | NA | 35 | <10 cases | NA | 73 108 | <10 cases | reference |
| Manitoba, Canada | 62 | 0% | NA | 0 | NA | NA | 14 365 | <10 cases | reference |
| Ontario, Canada | 303 | 0% | NA | 0 | NA | NA | 59 391 | <10 cases | reference |
| National, US | 19 884 | 0.07% | 2.80 (1.26 – 6.23) | 237 | <10 cases | NA | 20 121 | <10 cases | reference |
| COVID-19 diagnosis in trimester 3 |  |  |  |  |  |  |  |  |  |
| Tuscany, Italy | 297 | 0% | NA | 107 | 0% | NA | 1530 | 0% | reference |
| National, France | 0 | NA | NA | 218 | 0% | NA | 2772 | 0% | reference |
| Valencia, Spain | 957 | <10 cases | NA | 8 | 0% | NA | 3984 | 0% | reference |
| Aragon, Spain | 272 | 0% | NA | 11 | 0% | NA | 1347 | 0% | reference |
| National, Norway | 311 | 0% | NA | 128 | 0% | NA | 1539 | 0% | reference |
| National, Sweden | 2246 | <10 cases | NA | 499 | 0% | NA | 8255 | 0% | reference |
| Alberta, Canada | 691 | <10 cases | NA | 284 | <10 cases | NA | 65 565 | <10 cases | reference |
| Manitoba, Canada | 89 | 0% | NA | 65 | 0% | NA | 10 634 | <10 cases | reference |
| Ontario, Canada | 360 | 0% | NA | 0 | NA | NA | 32 126 | <10 cases | reference |
| National, US | 32 503 | <10 cases | 0.50 (0.20 – 1.22) | 1471 | 1.3% | 19.00 (2.81 – 128.70) | 33 978 | <10 cases | reference |

# **Table S9.** Prevalence of gestational diabetes and its association with COVID-19 in first trimester by hospitalization status

| Site | PREGNANT WOMEN WITH COVID-19 | | | | | | PREGNANT WOMEN WITHOUT COVID-19 | | |
| --- | --- | --- | --- | --- | --- | --- | --- | --- | --- |
|  | **Non-hospitalized cases** | | | **Hospitalized cases** | | |  |  |  |
|  | *Total N* | *Prevalence %* | *Adjusted RR (95% CI)* | *Total N* | *Prevalence %* | *Adjusted RR (95% CI)* | *Total N* | *Prevalence %* | *Adjusted RR (95% CI)* |
| COVID-19 diagnosis in trimester 1 |  |  |  |  |  |  |  |  |  |
| Tuscany, Italy | 149 | 10.7% | 1.04 (0.61 – 1.77) | <10 | <10 cases | 4.49 (1.09 – 18.58) | 603 | 7.8% | reference |
| National, France | 0 | NA | NA | <10 | <10 cases | 5.62 (0.56 – 56.04) | 18 | 5.6% | reference |
| Valencia, Spain | 1010 | 8.4% | 0.84 (0.67 – 1.06) | 11 | 0% | NA | 3804 | 7.2% | reference |
| Aragon, Spain | 156 | 9.0% | 0.68 (0.39 – 1.18) | <10 | 0% | NA | 552 | 11.6% | reference |
| National, Norway | 225 | 6.7% | 1.13 (0.65 – 1.97) | 18 | <10 cases | 0.88 (0.12 – 6.31) | 732 | 6.3% | reference |
| National, Sweden | 238 | <10 cases | 0.71 (0.33 – 1.53) | 26 | <10 cases | 1.23 (0.32 – 4.76) | 797 | 5.5% | reference |
| Alberta, Canada | 558 | 12.0% | 1.35 (1.08 – 1.69)* | <10 | <10 cases | 5.00 (2.41 – 10.38)* | 62 389 | 8.9% | reference |
| Manitoba, Canada | 13 | <10 cases | 0.76 (0.12 – 4.98) | 0 | NA | NA | 12 911 | 10.7% | reference |
| Ontario, Canada | 108 | <10 cases | 1.01 (0.52 – 1.97)* | 0 | NA | NA | 38 932 | 7.3% | reference |
| National, US | 15 751 | 12.6% | 0.99 (0.95 – 1.03) | 66 | 15.2% | 0.83 (0.47 – 1.47) | 15 817 | 12.9% | reference |

*Crude RR was used.

|  |
| --- |
|  |

# **Figure S2.** Gestational diabetes and its association with COVID-19 infection in first trimester by hospitalization status

# **Table S10.** Prevalence of pre-eclampsia and its association with COVID-19 in first trimester by hospitalization status

| Site | PREGNANT WOMEN WITH COVID-19 | | | | | | PREGNANT WOMEN WITHOUT COVID-19 | | |
| --- | --- | --- | --- | --- | --- | --- | --- | --- | --- |
|  | **Non-hospitalized cases** | | | **Hospitalized cases** | | |  |  |  |
|  | *Total N* | *Prevalence %* | *Adjusted RR (95% CI)* | *Total N* | *Prevalence %* | *Adjusted RR (95% CI)* | *Total N* | *Prevalence %* | *Adjusted RR (95% CI)* |
| COVID-19 diagnosis in trimester 1 |  |  |  |  |  |  |  |  |  |
| Tuscany, Italy | 149 | <10 cases | 0.88 (0.31 – 2.52) | <10 | 0% | NA | 603 | 2.3% | reference |
| Valencia, Spain | 1010 | 2.2% | 0.92 (0.56 – 1.50) | 11 | 0% | NA | 3804 | 1.7% | reference |
| Aragon, Spain | 156 | <10 cases | 0.58 (0.20 – 1.66) | <10 | 0% | NA | 552 | 3.8% | reference |
| National, Norway | 225 | <10 cases | 0.57 (0.20 – 1.64) | 18 | <10 cases | 1.53 (0.23 – 10.18) | 732 | 3.1% | reference |
| National, Sweden | 238 | 4.2% | 0.82 (0.41 – 1.65) | 26 | 0% | NA | 797 | 5.4% | reference |
| Alberta, Canada | 558 | 3.0% | 0.79 (0.49 – 1.26)* | <10 | <10 cases | 5.77 (1.70 – 19.59)* | 62 389 | 3.9% | reference |
| Manitoba, Canada | 13 | 0% | NA | 0 | NA | NA | 12 911 | 8.5% | reference |
| Ontario, Canada | 108 | <10 cases | 0.46 (0.07 – 3.23) | 0 | NA | NA | 38 932 | 2.1% | reference |
| National, US | 15 770 | 9.4% | 1.04 (0.99 – 1.09) | 67 | <10 cases | 0.75 (0.35 – 1.60) | 15 837 | 9.0% | reference |

*Crude RR was used.

|  |
| --- |
|  |

# **Figure S3.** Pre-eclamspia and its association with COVID-19 infection in first trimester by hospitalization status

# **Table S11.** Prevalence of caesarean section and its association with COVID-19 by trimester and hospitalization status

| Site | PREGNANT WOMEN WITH COVID-19 | | | | | | PREGNANT WOMEN WITHOUT COVID-19 | | |
| --- | --- | --- | --- | --- | --- | --- | --- | --- | --- |
|  | **Non-hospitalized cases** | | | **Hospitalized cases** | | |  |  |  |
|  | *Total N* | *Prevalence %* | *Adjusted RR (95% CI)* | *Total N* | *Prevalence %* | *Adjusted RR (95% CI)* | *Total N* | *Prevalence %* | *Adjusted RR (95% CI)* |
| COVID-19 diagnosis in trimester 1 |  |  |  |  |  |  |  |  |  |
| Tuscany, Italy | 177 | 18.1% | 0.98 (0.69 – 1.40) | <10 | 0% | NA | 603 | 18.2% | reference |
| National, France | 0 | NA | NA | <6 | 0% | NA | 18 | <10 cases | reference |
| Valencia, Spain | 1231 | 7.8% | 0.93 (0.74 – 1.16) | 13 | <10 cases | 1.84 (0.51 – 6.58) | 3804 | 8.4% | reference |
| Alberta, Canada | 483 | 34.0% | 1.06 (0.94 – 1.20)* | <10 | <10 cases | 2.08 (1.31 – 3.31)* | 56 542 | 32.0% | reference |
| Manitoba, Canada | <10 | 0% | NA | 0 | NA | NA | 10 681 | 26.8% | reference |
| Ontario, Canada | 65 | 18.5% | 0.88 (0.53 – 1.46) | 0 | NA | NA | 32 244 | 21.1% | reference |
| National, US | 15 773 | 21.8% | 0.99 (0.97 – 1.02) | 67 | 41.8% | 1.27 (0.92 – 1.77) | 15 840 | 22.0% | reference |
| COVID-19 diagnosis in trimester 2 |  |  |  |  |  |  |  |  |  |
| Tuscany, Italy | 257 | 26.8% | 1.54 (1.20 – 1.98) | 17 | 17.6% | 1.02 (0.36 – 2.91) | 822 | 17.3% | reference |
| National, France | 0 | NA | NA | 85 | 18.8% | 1.15 (0.69 – 1.91) | 255 | 16.9% | reference |
| Valencia, Spain | 1032 | 10.1% | 1.17 (0.94 – 1.45) | 12 | <10 cases | 2.05 (0.57 – 7.38) | 3141 | 8.6% | reference |
| Alberta, Canada | 651 | 34.4% | 1.09 (0.98 – 1.21)* | 27 | 37.0% | 1.17 (0.72 – 1.92)* | 65 903 | 31.6% | reference |
| Manitoba, Canada | 45 | 24.4% | 0.88 (0.53 – 1.45) | 0 | NA | NA | 10 681 | 26.8% | reference |
| Ontario, Canada | 179 | 22.3% | 1.09 (0.83 – 1.43) | 0 | NA | NA | 32 244 | 21.1% | reference |
| National, US | 19 862 | 21.3% | 0.98 (0.96 – 1.01) | 234 | 27.4% | 1.28 (1.02 – 1.61) | 20 096 | 21.7% | reference |
| COVID-19 diagnosis in trimester 3 |  |  |  |  |  |  |  |  |  |
| Tuscany, Italy | 297 | 21.5% | 1.20 (0.94 – 1.54) | 107 | 23.4% | 1.30 (0.91 – 1.86) | 1530 | 17.8% | reference |
| National, France | 0 | NA | NA | 218 | 31.7% | 1.54 (1.25 – 1.89) | 2772 | 19.9% | reference |
| Valencia, Spain | 957 | 13.6% | 1.54 (1.27 – 1.86) | <10 | 0% | NA | 3984 | 8.8% | reference |
| Alberta, Canada | 691 | 30.2% | 0.96 (0.85 – 1.07)* | 284 | 39.8% | 1.26 (1.09 – 1.45)* | 65 547 | 31.6% | reference |
| Manitoba, Canada | 89 | 29.2% | 1.09 (0.79 – 1.51)* | 65 | 29.2% | 1.09 (0.75 – 1.60)* | 10 634 | 26.8% | reference |
| Ontario, Canada | 360 | 19.4% | 0.94 (0.76 – 1.16) | 0 | NA | NA | 32 126 | 21.0% | reference |

*Crude RR was used.

|  |
| --- |
|  |

# **Figure S4.** Caesarean section and its association with COVID-19 infection in first trimester by hospitalization status

|  |
| --- |
|  |

# **Figure S5.** Caesarean section and its association with COVID-19 infection in second trimester by hospitalization status

|  |
| --- |
|  |

# **Figure S6.** Caesarean section and its association with COVID-19 infection in third trimester by hospitalization status

# **Table S12.** Prevalence of preterm birth and its association with COVID-19 by trimester and hospitalization status

| Site | PREGNANT WOMEN WITH COVID-19 | | | | | | PREGNANT WOMEN WITHOUT COVID-19 | | |
| --- | --- | --- | --- | --- | --- | --- | --- | --- | --- |
|  | **Non-hospitalized cases** | | | **Hospitalized cases** | | |  |  |  |
|  | *Total N* | *Prevalence %* | *Adjusted RR (95% CI)* | *Total N* | *Prevalence %* | *Adjusted RR (95% CI)* | *Total N* | *Prevalence %* | *Adjusted RR (95% CI)* |
| COVID-19 diagnosis in trimester 1 |  |  |  |  |  |  |  |  |  |
| Tuscany, Italy | 132 | <10 cases | 0.76 (0.34 – 1.68) | 1 | 0% | NA | 433 | 6.9% | reference |
| National, France | 0 | NA | NA | 6 | 0% | NA | 17 | 0% | reference |
| Valencia, Spain | 750 | 8.1% | 1.02 (0.77 – 1.35) | 8 | <10 cases | 1.55 (0.24 – 9.88) | 2354 | 8.0% | reference |
| Aragon, Spain | 138 | 7.2% | 1.52 (0.72 – 3.17) | 2 | 0% | NA | 434 | 4.8% | reference |
| National, Norway | 225 | 3.1% | 0.60 (0.27 – 1.33) | 18 | 16.7% | 2.72 (0.94 – 7.89) | 722 | 5.4% | reference |
| National, Sweden | 238 | 4.2% | 0.88 (0.43 – 1.77) | 26 | 0% | NA | 794 | 4.9% | reference |
| Alberta, Canada | 483 | 8.5% | 0.97 (0.72 – 1.30)* | <10 | <10 cases | 3.80 (1.51 – 9.58)* | 56 542 | 8.8% | reference |
| Manitoba, Canada | <10 | 0% | NA | 0 | NA | NA | 10 681 | 9.0% | reference |
| Ontario, Canada | 65 | 15.4% | 1.68 (0.95 – 2.97) | 0 | NA | NA | 32 244 | 9.4% | reference |
| National, US | 15 771 | 5.3% | 0.99 (0.93 – 1.06) | 67 | 16.4% | 2.20 (1.04 – 4.64) | 15 838 | 5.3% | reference |
| COVID-19 diagnosis in trimester 2 |  |  |  |  |  |  |  |  |  |
| Tuscany, Italy | 237 | 4.6% | 1.05 (0.53 – 2.08) | 16 | <10 cases | 2.89 (0.76 – 11.04) | 596 | 4.4% | reference |
| National, France | 0 | NA | NA | 85 | <10 cases | 1.16 (0.50 – 2.66) | 247 | 7.7% | reference |
| Valencia, Spain | 780 | 6.8% | 0.90 (0.66 – 1.21) | 12 | 0% | NA | 1877 | 7.7% | reference |
| Aragon, Spain | 233 | <10 cases | 0.82 (0.40 – 1.68) | <10 | 0% | NA | 615 | 4.9% | reference |
| National, Norway | 367 | 3.5% | 0.70 (0.39 – 1.26) | 48 | <10 cases | 1.23 (0.73 – 4.47) | 1236 | 5.2% | reference |
| National, Sweden | 1256 | 5.5% | 0.88 (0.67 – 1.15) | 221 | 8.6% | 2.14 (1.19 – 3.86) | 4502 | 5.9% | reference |
| Alberta, Canada | 651 | 10.0% | 1.22 (0.97 – 1.54)* | 27 | <10 cases | 1.81 (0.73 – 4.47)* | 65 903 | 8.2% | reference |
| Manitoba, Canada | 45 | <10 cases | 0.89 (0.36 – 2.21) | 0 | NA | NA | 10 681 | 9.0% | reference |
| Ontario, Canada | 179 | 10.6% | 1.21 (0.74 – 1.72) | 0 | NA | NA | 32 244 | 9.4% | reference |
| National, US | 19 857 | 5.3% | 1.00 (0.95 – 1.06) | 233 | 9.9% | 1.35 (0.89 – 2.05) | 20 090 | 5.3% | reference |
| COVID-19 diagnosis in trimester 3 |  |  |  |  |  |  |  |  |  |
| Tuscany, Italy | 194 | 5.2% | 0.61 (0.32 – 1.18) | 43 | <10 cases | 3.77 (1.99 – 7.11) | 1086 | 5.5% | reference |
| National, France | 0 | NA | NA | 130 | 10.8% | 1.59 (0.95 – 2.65) | 2719 | 6.7% | reference |
| Valencia, Spain | 620 | 7.3% | 0.60 (0.44 – 0.82) | <10 | <10 cases | 3.61 (1.09 – 11.89) | 2462 | 7.8% | reference |
| Aragon, Spain | 182 | <10 cases | 0.63 (0.30 – 1.32) | <10 | <10 cases | 4.07 (0.70 – 23.70) | 1090 | 4.6% | reference |
| National, Norway | 266 | 5.6% | 0.89 (0.52 – 1.51) | 60 | <10 cases | 1.20 (0.45 – 3.18) | 1528 | 5.4% | reference |
| National, Sweden | 2239 | 4.7% | 1.85 (1.45 – 2.36) | 497 | 9.9% | 3.01 (2.02 – 4.49) | 8241 | 2.6% | reference |
| Alberta, Canada | 691 | 5.8% | 0.75 (0.56 – 1.02)* | 284 | 19.7% | 2.56 (2.02 – 3.24)* | 65 547 | 7.7% | reference |
| Manitoba, Canada | 89 | <10 cases | 1.18 (0.63 – 2.20)* | 65 | 26.2% | 3.06 (2.02 – 4.63)* | 10 634 | 8.5% | reference |
| Ontario, Canada | 360 | 8.6% | 0.94 (0.67 – 1.31) | 0 | NA | NA | 32 126 | 9.1% | reference |

*Crude RR was used.

|  |
| --- |
|  |

# **Figure S7.** Preterm birth and its association with COVID-19 infection in first trimester by hospitalization status

|  |
| --- |
|  |

# **Figure S8.** Preterm birth and its association with COVID-19 infection in second trimester by hospitalization status

|  |
| --- |
|  |

# **Figure S9.** Preterm birth and its association with COVID-19 infection in third trimester by hospitalization status

# **Table S13.** Prevalence of stillbirth and its association with COVID-19 by trimester and hospitalization status

| Site | PREGNANT WOMEN WITH COVID-19 | | | | | | PREGNANT WOMEN WITHOUT COVID-19 | | |
| --- | --- | --- | --- | --- | --- | --- | --- | --- | --- |
|  | **Non-hospitalized cases** | | | **Hospitalized cases** | | |  |  |  |
|  | *Total N* | *Prevalence %* | *Adjusted RR (95% CI)* | *Total N* | *Prevalence %* | *Adjusted RR (95% CI)* | *Total N* | *Prevalence %* | *Adjusted RR (95% CI)* |
| COVID-19 diagnosis in trimester 1 |  |  |  |  |  |  |  |  |  |
| Tuscany, Italy | 149 | <10 cases | 3.21 (0.20 – 52.14) | <10 | 0% | NA | 466 | <10 cases | reference |
| National, France | 0 | NA | NA | <10 | 0% | NA | 17 | 0% | reference |
| Valencia, Spain | 1006 | <10 cases | 0.58 (0.20 – 1.73) | 11 | 0% | NA | 2940 | 0.6% | reference |
| Aragon, Spain | 156 | 0% | NA | <10 | 0% | NA | 475 | <10 cases | reference |
| National, Norway | 225 | 0% | NA | 18 | 0% | NA | 722 | 0% | reference |
| National, Sweden | 238 | 0% | NA | 26 | 0% | NA | 797 | <10 cases | reference |
| Alberta, Canada | 483 | <10 cases | 0.81 (0.20 – 3.23)* | <10 | 0% | NA | 56 542 | 0.5% | reference |
| Manitoba, Canada | <10 | 0% | NA | 0 | NA | NA | 10 681 | 0.8% | reference |
| Ontario, Canada | 65 | 0% | NA | 0 | NA | NA | 32 244 | 0.04% | reference |
| COVID-19 diagnosis in trimester 2 |  |  |  |  |  |  |  |  |  |
| Tuscany, Italy | 257 | <10 cases | NA | 17 | 0% | NA | 642 | 0% | reference |
| National, France | 0 | NA | NA | 85 | <10 cases | NA | 253 | <10 cases | reference |
| Valencia, Spain | 1022 | <10 cases | 0.95 (0.30 – 3.04) | 12 | 0% | NA | 2377 | <10 cases | reference |
| Aragon, Spain | 250 | <10 cases | NA | <10 | <10 cases | NA | 661 | 0% | reference |
| National, Norway | 368 | <10 cases | 1.24 (0.13 – 12.04) | 48 | 0% | NA | 1238 | <10 cases | reference |
| National, Sweden | 1256 | 0% | NA | 223 | <10 cases | 6.42 (0.58 – 70.81) | 4515 | 0.3% | reference |
| Alberta, Canada | 651 | <10 cases | 0.66 (0.17 – 2.66)* | 27 | <10 cases | 16.01 (4.20 – 61.02)* | 65 903 | 0.5% | reference |
| Manitoba, Canada | 45 | <10 cases | 2.44 (0.36 – 16.68) | 0 | NA | NA | 10 681 | 0.8% | reference |
| Ontario, Canada | 179 | 0% | NA | 0 | NA | NA | 32 244 | 0.04% | reference |
| COVID-19 diagnosis in trimester 3 |  |  |  |  |  |  |  |  |  |
| Tuscany, Italy | 297 | 0% | NA | 107 | 0% | NA | 1187 | <10 cases | reference |
| National, France | 0 | NA | NA | 218 | <10 cases | NA | 2735 | 0.4% | reference |
| Valencia, Spain | 957 | <10 cases | 0.20 (0.03 – 1.51) | <10 | 0% | NA | 3122 | 0.4% | reference |
| Aragon, Spain | 272 | <10 cases | 9.73 (1.78 – 53.17) | 11 | 0% | NA | 1175 | <10 cases | reference |
| National, Norway | 311 | <10 cases | 10.13 (0.92 – 112.03) | 128 | <10 cases | NA | 1529 | <10 cases | reference |
| National, Sweden | 2246 | <10 cases | 1.75 (0.69 – 4.46) | 499 | <10 cases | 3.09 (0.44 – 21.95) | 8255 | 0.2% | reference |
| Alberta, Canada | 691 | <10 cases | 2.04 (0.76 – 5.48)* | 284 | 0% | NA | 65 547 | 0.3% | reference |
| Manitoba, Canada | 89 | 0% | NA | 65 | <10 cases | 7.33 (2.36 – 22.69) | 10 634 | 0.6% | reference |
| Ontario, Canada | 360 | <10 cases | NA | 0 | NA | NA | 32 126 | <10 cases | reference |

*Crude RR was used.

|  |
| --- |

# **Figure S10.** Stillbirth and its association with COVID-19 infection in second trimester among non-hospitalized pregnant women

|  |
| --- |
|  |

# **Figure S11.** Stillbirth and its association with COVID-19 infection in third trimester by hospitalization status

# **Table S14.** Prevalence of neonatal death and its association with COVID-19 by trimester and hospitalization status

| Site | PREGNANT WOMEN WITH COVID-19 | | | | | | PREGNANT WOMEN WITHOUT COVID-19 | | |
| --- | --- | --- | --- | --- | --- | --- | --- | --- | --- |
|  | **Non-hospitalized cases** | | | **Hospitalized cases** | | |  |  |  |
|  | *Total N* | *Prevalence %* | *Adjusted RR (95% CI)* | *Total N* | *Prevalence %* | *Adjusted RR (95% CI)* | *Total N* | *Prevalence %* | *Adjusted RR (95% CI)* |
| COVID-19 diagnosis in trimester 1 |  |  |  |  |  |  |  |  |  |
| Tuscany, Italy | 132 | 0% | NA | <10 | 0% | NA | 438 | <10 cases | reference |
| Valencia, Spain | 667 | 0% | NA | <10 | 0% | NA | 2106 | <10 cases | reference |
| Aragon, Spain | 86 | 0% | NA | <10 | 0% | NA | 303 | 0% | reference |
| National, Norway | 219 | <10 cases | NA | 19 | 0% | NA | 707 | 0% | reference |
| National, Sweden | 238 | 0% | NA | 26 | 0% | NA | 794 | 0% | reference |
| Manitoba, Canada | <10 | 0% | NA | 0 | NA | NA | 10 594 | 0.3% | reference |
| Ontario, Canada | 65 | 0% | NA | 0 | NA | NA | 32 244 | 0.3% | reference |
| COVID-19 diagnosis in trimester 2 |  |  |  |  |  |  |  |  |  |
| Tuscany, Italy | 237 | 0% | NA | 16 | 0% | NA | 591 | 0% | reference |
| Valencia, Spain | 685 | 0% | NA | <10 | 0% | NA | 1672 | <10 cases | reference |
| Aragon, Spain | 153 | 0% | NA | <10 | 0% | NA | 452 | <10 cases | reference |
| National, Norway | 364 | <10 cases | NA | 51 | 0% | NA | 1212 | 0% | reference |
| National, Sweden | 1256 | <10 cases | 0.76 (0.09 – 6.84) | 221 | 0% | NA | 4502 | <10 cases | reference |
| Manitoba, Canada | 44 | <10 cases | 8.92 (1.24 – 64.19)* | 0 | NA | NA | 10 594 | 0.3% | reference |
| Ontario, Canada | 179 | 0% | NA | 0 | NA | NA | 32 244 | 0.04% | reference |
| COVID-19 diagnosis in trimester 3 |  |  |  |  |  |  |  |  |  |
| Tuscany, Italy | 271 | 0% | NA | 101 | 0% | NA | 1071 | <10 cases | reference |
| Valencia, Spain | 698 | 0% | NA | <10 | 0% | NA | 2211 | <10 cases | reference |
| Aragon, Spain | 186 | 0% | NA | <10 | 0% | NA | 809 | 0% | reference |
| National, Norway | 300 | 0% | NA | 130 | 0% | NA | 1505 | 0% | reference |
| National, Sweden | 2239 | 0% | NA | 497 | <10 cases | NA | 8241 | <10 cases | reference |
| Manitoba, Canada | 89 | <10 cases | 19.79 (4.49 – 87.14) | 62 | 0% | NA | 10 567 | 0.1% | reference |
| Ontario, Canada | 360 | 0% | NA | 0 | NA | NA | 32 126 | 0.1% | reference |

*Crude RR was used.

# **Table S15.** Prevalence of low birth weight and its association with COVID-19 by trimester and hospitalization status

| Site | PREGNANT WOMEN WITH COVID-19 | | | | | | PREGNANT WOMEN WITHOUT COVID-19 | | |
| --- | --- | --- | --- | --- | --- | --- | --- | --- | --- |
|  | **Non-hospitalized cases** | | | **Hospitalized cases** | | |  |  |  |
|  | *Total N* | *Prevalence %* | *Adjusted RR (95% CI)* | *Total N* | *Prevalence %* | *Adjusted RR (95% CI)* | *Total N* | *Prevalence %* | *Adjusted RR (95% CI)* |
| COVID-19 diagnosis in trimester 1 |  |  |  |  |  |  |  |  |  |
| Tuscany, Italy | 132 | <10 cases | 0.73 (0.37 – 1.46) | <10 | 0% | NA | 438 | 9.4% | reference |
| Valencia, Spain | 681 | 7.5% | 0.93 (0.68 – 1.26) | <10 | 0% | NA | 1972 | 8.2% | reference |
| Aragon, Spain | 86 | <10 cases | 1.17 (0.52 – 2.65) | <10 | 0% | NA | 303 | 6.9% | reference |
| National, Norway | 219 | 5.0% | 1.15 (0.59 – 2.24) | 19 | <10 cases | 3.18 (1.14 – 8.92) | 707 | 4.4% | reference |
| National, Sweden | 238 | <10 cases | 0.46 (0.16 – 1.33) | 26 | <10 cases | 1.47 (0.13 – 16.84) | 797 | 3.5% | reference |
| Alberta, Canada | 481 | 6.4% | 0.89 (0.63 – 1.26)* | <10 | <10 cases | 3.08 (0.91 – 10.45)* | 56 283 | 7.2% | reference |
| Manitoba, Canada | <10 | 0% | NA | 0 | NA | NA | 10 594 | 5.4% | reference |
| Ontario, Canada | 65 | 16.9% | 2,21 (1.29 – 3.79) | 0 | NA | NA | 32 244 | 7.8% | reference |
| National, US | 4291 | 2.2% | 0.80 (0.61 – 1,04) | 19 | 0% | NA | 4310 | 2.2% | reference |
| COVID-19 diagnosis in trimester 2 |  |  |  |  |  |  |  |  |  |
| Tuscany, Italy | 237 | 6.8% | 1.10 (0.62 – 1.96) | 16 | <10 cases | 2.05 (0.57 – 7.38) | 591 | 6.1% | reference |
| Valencia, Spain | 693 | 6.8% | 0.86 (0.63 – 1.18) | <10 | 0% | NA | 1824 | 7.9% | reference |
| Aragon, Spain | 153 | 7.2% | 0.81 (0.43 – 1.52) | <10 | 0% | NA | 452 | 9.5% | reference |
| National, Norway | 364 | 3.0% | 0.63 (0.33 – 1.18) | 51 | <10 cases | 1.99 (0.83 – 4.73) | 1212 | 4.9% | reference |
| National, Sweden | 1256 | 2.8% | 0.72 (0.50 – 1.03) | 223 | 7.6% | 3.39 (1.71 – 6.72) | 4514 | 3.7% | reference |
| Alberta, Canada | 649 | 7.6% | 1.12 (0.85 – 1.46)* | 26 | <10 cases | 1.14 (0.30 – 4.30)* | 65 629 | 6.8% | reference |
| Manitoba, Canada | 44 | <10 cases | 0.84 (0.22 – 3.26) | 0 | NA | NA | 10 594 | 5.4% | reference |
| Ontario, Canada | 179 | 8.4% | 1.07 (0.66 – 1.74) | 0 | NA | NA | 32 244 | 7.8% | reference |
| National, US | 6225 | 2.2% | 0.95 (0.76 – 1.19) | 73 | <10 cases | 2.33 (0.89 – 6.13) | 6298 | 2.3% | reference |
| COVID-19 diagnosis in trimester 3 |  |  |  |  |  |  |  |  |  |
| Tuscany, Italy | 271 | 6.3% | 0.77 (0.46 – 1.27) | 101 | <10 cases | 0.97 (0.48 – 1.94) | 1071 | 8.2% | reference |
| Valencia, Spain | 635 | 6.8% | 0.69 (0.51 – 0.94) | <10 | 0% | NA | 2558 | 9.7% | reference |
| Aragon, Spain | 186 | <10 cases | 0.60 (0.30 – 1.18) | <10 | <10 cases | 1.78 (0.28 – 11.46) | 809 | 7.7% | reference |
| National, Norway | 300 | 4.7% | 1.25 (0.71 – 2.21) | 130 | <10 cases | 0.82 (0.31 – 2.19) | 1505 | 3.7% | reference |
| National, Sweden | 2246 | 3.3% | 2.30 (1.69 – 3.12) | 499 | 6.4% | 3.05 (1.86 – 5.00) | 8255 | 1.5% | reference |
| Alberta, Canada | 687 | 4.7% | 0.74 (0.53 – 1.04)* | 284 | 12.3% | 1.96 (1.43 – 2.67)* | 65 385 | 6.3% | reference |
| Manitoba, Canada | 89 | <10 cases | 1.09 (0.46 – 2.56)* | 62 | 24.2% | 4.68 (2.99 – 7.33)* | 10 567 | 5.2% | reference |
| Ontario, Canada | 360 | 7.8% | 1.03 (0.72 – 1.48) | 0 | NA | NA | 32 126 | 7.4% | reference |
| National, US | 11 782 | 1.8% | 0.87 (0.73 – 1.04) | 492 | 8.9% | 4.24 (3.12 – 5.76) | 12 274 | 2.1% | reference |

*Crude RR was used.

|  |
| --- |
|  |

# **Figure S12.** Low birth weight and its association with COVID-19 infection in first trimester by hospitalization status

|  |
| --- |
|  |

# **Figure S13.** Low birth weight and its association with COVID-19 infection in second trimester by hospitalization status

|  |
| --- |
|  |

# **Figure S14.** Low birth weight and its association with COVID-19 infection in third trimester by hospitalization status

# **Table S16.** Prevalence of small for gestational age and its association with COVID-19 by trimester and hospitalization status

| Site | PREGNANT WOMEN WITH COVID-19 | | | | | | PREGNANT WOMEN WITHOUT COVID-19 | | |
| --- | --- | --- | --- | --- | --- | --- | --- | --- | --- |
|  | **Non-hospitalized cases** | | | **Hospitalized cases** | | |  |  |  |
|  | *Total N* | *Prevalence %* | *Adjusted RR (95% CI)* | *Total N* | *Prevalence %* | *Adjusted RR (95% CI)* | *Total N* | *Prevalence %* | *Adjusted RR (95% CI)* |
| COVID-19 diagnosis in trimester 1 |  |  |  |  |  |  |  |  |  |
| Tuscany, Italy | 132 | <10 cases | 0.61 (0.21 – 1.75) | <10 | 0% | NA | 438 | 5.0% | reference |
| Aragon, Spain | 86 | <10 cases | 6.92 (0.67 – 71.54) | <10 | 0% | NA | 303 | <10 cases | reference |
| National, Norway | 219 | <10 cases | 1.40 (0.46 – 1.53) | 10 | 0% | NA | 707 | 2.8% | reference |
| National, Sweden | 217 | <10 cases | 1.10 (0.40 – 3.05) | 23 | <10 cases | 1.56 (0.14 – 17.42) | 756 | 2.1% | reference |
| Alberta, Canada | 481 | 10.4% | 1.02 (0.78 – 1.33)* | <10 | 0% | NA | 56 283 | 10.2% | reference |
| Manitoba, Canada | <10 | 0% | NA | 0 | NA | NA | 10 594 | 2.5% | reference |
| Ontario, Canada | 65 | 20.0% | 1.93 (1.19 – 3.13) | 0 | NA | NA | 32 244 | 10.4% | reference |
| National, US | 4291 | 2.9% | 1.07 (0.83 – 1.37) | 19 | 0% | NA | 4310 | 2.7% | reference |
| COVID-19 diagnosis in trimester 2 |  |  |  |  |  |  |  |  |  |
| Tuscany, Italy | 237 | <10 cases | 0.95 (0.43 – 2.10) | 16 | <10 cases | 1.80 (0.25 – 12.86) | 591 | 3.6% | reference |
| Aragon, Spain | 153 | <10 cases | 1.41 (0.44 – 4.49) | <10 | 0% | NA | 452 | <10 cases | reference |
| National, Norway | 364 | <10 cases | 0.43 (0.15 – 1.18) | 51 | <10 cases | 0.76 (0.11 – 5.42) | 1212 | 2.6% | reference |
| National, Sweden | 1129 | 1.4% | 0.68 (0.39 – 1.16) | 201 | <10 cases | 1.15 (0.42 – 3.20) | 4045 | 2.1% | reference |
| Alberta, Canada | 649 | 12.2% | 1.20 (0.98 – 1.48)* | 26 | <10 cases | 0.76 (0.20 – 2.88)* | 65 629 | 10.1% | reference |
| Manitoba, Canada | 44 | 0% | NA | 0 | NA | NA | 10 594 | 2.5% | reference |
| Ontario, Canada | 179 | 7.8% | 0.75 (0.45 – 1.24) | 0 | NA | NA | 32 244 | 10.4% | reference |
| National, US | 6225 | 2.8% | 0.97 (0.79 – 1.19) | 73 | <10 cases | 0.47 (0.07 – 3.28) | 6298 | 2.9% | reference |
| COVID-19 diagnosis in trimester 3 |  |  |  |  |  |  |  |  |  |
| Tuscany, Italy | 271 | 6.3% | 1.32 (0.77 – 2.25) | 101 | <10 cases | 0.62 (0.20 – 1.95) | 1071 | 4.8% | reference |
| Aragon, Spain | 186 | <10 cases | 0.80 (0.17 – 3.69) | <10 | <10 cases | 9.08 (1.48 – 55.83) | 809 | 1.4% | reference |
| National, Norway | 300 | <10 cases | 1.21 (0.46 – 3.16) | 130 | <10 cases | 2.17 (0.78 – 6.08) | 1505 | 1.4% | reference |
| National, Sweden | 2118 | 2.0% | 1.21 (0.85 – 1.73) | 472 | <10 cases | 0.64 (0.28 – 1.44) | 7824 | 1.8% | reference |
| Alberta, Canada | 687 | 7.9% | 0.79 (0.61 – 1.02)* | 284 | 6.3% | 0.64 (0.41 – 0.99)* | 65 385 | 10.0% | reference |
| Manitoba, Canada | 89 | <10 cases | 2.26 (0.96 – 5.33)* | 62 | <10 cases | 2.59 (1.00 – 6.74)* | 10 567 | 2.5% | reference |
| Ontario, Canada | 360 | 11.1% | 1.08 (0.81 – 1.45) | 0 | NA | NA | 32 126 | 10.3% | reference |
| National, US | 11 782 | 2.8% | 0.99 (0.85 – 1.15) | 492 | 3.0% | 1.08 (0.65 – 1.79) | 12 274 | 2.8% | reference |

*Crude RR was used.

|  |
| --- |

# **Figure S15.** Small for gestational age and its association with COVID-19 infection in first trimester among non-hospitalized pregnant women

|  |
| --- |
|  |

# **Figure S16.** Small for gestational age and its association with COVID-19 infection in second trimester by hospitalization status

|  |
| --- |
|  |

# **Figure S17.** Small for gestational age and its association with COVID-19 infection in third trimester by hospitalization status

# **Table S17.** Prevalence of major congenital abnormalities and its association with COVID-19 in first trimester by hospitalization status

| Site | PREGNANT WOMEN WITH COVID-19 | | | | | | PREGNANT WOMEN WITHOUT COVID-19 | | |
| --- | --- | --- | --- | --- | --- | --- | --- | --- | --- |
|  | **Non-hospitalized cases** | | | **Hospitalized cases** | | |  |  |  |
|  | *Total N* | *Prevalence %* | *Adjusted RR (95% CI)* | *Total N* | *Prevalence %* | *Adjusted RR (95% CI)* | *Total N* | *Prevalence %* | *Adjusted RR (95% CI)* |
| COVID-19 diagnosis in trimester 1 |  |  |  |  |  |  |  |  |  |
| Tuscany, Italy | 132 | 0% | NA | <10 | 0% | NA | 438 | 0% | reference |
| Aragon, Spain | 86 | 0% | NA | <10 | 0% | NA | 303 | 0% | reference |
| National, Norway | 219 | <10 cases | 1.28 (0.54 – 3.02) | 19 | <10 cases | 2.10 (0.31 – 14.35) | 707 | 2.5% | reference |
| National, Sweden | 238 | 6.3% | 1.15 (0.63 – 2.08) | 26 | <10 cases | 0.49 (0.06 – 4.13) | 794 | 5.7% | reference |
| Alberta, Canada | 481 | 3.5% | 0.92 (0.57 – 1.46)* | <10 | <10 cases | 2.88 (0.45 – 18.26)* | 56 283 | 3.9% | reference |
| Manitoba, Canada | <10 | 0% | NA | 0 | NA | NA | 10 594 | 5.5% | reference |
| Ontario, Canada | 65 | 15.4% | 2.08 (1.18 – 3.69) | 0 | NA | NA | 32 244 | 7.4% | reference |
| National, US | 4291 | 11.0% | 1.05 (0.93 – 1.19) | 19 | <10 cases | 1.01 (0.27 – 3.77) | 4310 | 11.0% | reference |

*Crude RR was used.

|  |
| --- |
|  |

# **Figure S18.** Major congenital abnormalties and its association with COVID-19 infection in first trimester by hospitalization status

1. Patwardhan M, Eckert LO, Spiegel H, et al. Maternal death: Case definition and guidelines for data collection, analysis, and presentation of immunization safety data. *Vaccine*. 2016;34(49):6077. [↑](#footnote-ref-2)
2. Rouse CE, Eckert LO, Wylie BJ, et al. Hypertensive disorders of pregnancy: case definitions & guidelines for data collection, analysis, and presentation of immunization safety data. *Vaccine*. 2016;34(49):6069. [↑](#footnote-ref-3)
3. Kachikis A, Eckert LO, Walker C, et al. Gestational diabetes mellitus: Case definition & guidelines for data collection, analysis, and presentation of immunization safety data. *Vaccine*. 2017;35(48Part A):6555. [↑](#footnote-ref-4)
4. Quinn JA, Munoz FM, Gonik B, et al. Preterm birth: Case definition & guidelines for data collection, analysis, and presentation of immunisation safety data. *Vaccine*. 2016;34(49):6047-6056. [↑](#footnote-ref-5)
5. Da Silva FT, Gonik B, McMillan M, et al. Stillbirth: Case definition and guidelines for data collection, analysis, and presentation of maternal immunization safety data. *Vaccine*. 2016;34(49):6057. doi:10.1016/J.VACCINE.2016.03.044 [↑](#footnote-ref-6)
6. Pathirana J, Muñoz FM, Abbing-Karahagopian V, et al. Neonatal death: Case definition & guidelines for data collection, analysis, and presentation of immunization safety data. *Vaccine*. 2016;34(49):6027-6037. [↑](#footnote-ref-7)
7. Cutland CL, Lackritz EM, Mallett-Moore T, et al. Low birth weight: Case definition & guidelines for data collection, analysis, and presentation of maternal immunization safety data. *Vaccine*. 2017;35(48Part A):6492. [↑](#footnote-ref-8)
8. Schlaudecker EP, Munoz FM, Bardají A, et al. Small for gestational age: Case definition & guidelines for data collection, analysis, and presentation of maternal immunisation safety data. *Vaccine*. 2017;35(48Part A):6518. doi:10.1016/J.VACCINE.2017.01.040 [↑](#footnote-ref-9)
9. DeSilva M, Munoz FM, Mcmillan M, et al. Congenital anomalies: Case definition and guidelines for data collection, analysis, and presentation of immunization safety data. *Vaccine*. 2016;34(49):6015. [↑](#footnote-ref-10)
